# Progress and Future Potential of All-Small-Molecule Organic Solar Cells Based on the Benzodithiophene Donor Material

**DOI:** 10.3390/molecules28073171

**Published:** 2023-04-02

**Authors:** Shabaz Alam, Jaewon Lee

**Affiliations:** Department of Chemical Engineering and Applied Chemistry, Chungnam National University, Daejeon 34134, Republic of Korea; shabaz@cnu.ac.kr

**Keywords:** organic solar cells, bulk heterojunction, benzodithiophene, non-fullerene acceptor, morphology, stability

## Abstract

Organic solar cells have obtained a prodigious amount of attention in photovoltaic research due to their unique features of light weight, low cost, eco-friendliness, and semitransparency. A rising trend in this field is the development of all-small-molecules organic solar cells (ASM-OSCs) due to their merits of excellent batch-to-batch reproducibility, well-defined structures, and simple purification. Among the numerous organic photovoltaic (OPV) materials, benzodithiophene (BDT)-based small molecules have come to the fore in achieving outstanding power conversion efficiency (PCE) and breaking the 17% efficiency barrier in single-junction OPV devices, indicating the significant potential of this class of materials in commercial photovoltaic applications. This review specially focuses on up-to-date information about improvements in BDT-based ASM-OSCs since 2011 and provides an outlook on the most significant challenges that remain in the field. We believe there will be more exciting BDT-based photovoltaic materials and devices developed in the near future.

## 1. Introduction

The quality of modern life depends heavily on reliable, plentiful, and economical energy. Overreliance on fossil fuels is damaging to human health and the environment. Under these circumstances, providing affordable, safe, and clean energy is one of the major challenges in the scientific community. Solution-processed bulk-heterojunction (BHJ) organic solar cells (OSCs) have emerged as a potential contender for next-generation photovoltaic technology because of their advantages, such as low carbon footprint, low-temperature processing, short energy payback period, and facile manufacture into flexible, lightweight, and semitransparent products. Power conversion efficiencies (PCEs) have exceeded 19% in single-junction OSCs based on conjugated polymers as electron donor materials in recent years, thanks to the design of non-fullerene acceptors (NFAs) and device optimization [1,2,3,4,5]. Meanwhile, solution-processed small-molecule-based OSCs (SM-OSCs) are emerging as a competitive alternative to their polymer counterparts due to several important advantages of small molecules, such as well-defined structures and therefore less batch-to-batch variation, easier band structure control, etc. Recently, several solution-processed SM-OSCs have demonstrated PCEs exceeding 15%, which is closing the performance gap with the most promising polymer-based OSCs and may demonstrate even more potential for OSC technologies [6,7,8]. 

Benzo[1,2-b:4,5-b′]dithiophene (BDT) is the most attractive electron-donating unit and widely used chemical moiety in organic solar cells [9,10,11]. The BDT unit contains a symmetric and planar conjugated structure that causes a strong intermolecular orbital overlap, benefits the electron delocalization and π-π stacking in solid-state thin film, and leads to efficient charge transport in the device. In the past decade, huge numbers of conjugated small molecular donors based on the BDT unit have been developed, focusing on lateral side chain engineering, backbone engineering (using different building blocks), and end-group acceptor engineering using different acceptor moieties containing heteroatoms (such as F, O, CN, etc.). The symmetrical and planar conjugated molecular backbone of the A-π-D-π-A-types with a central BDT unit could facilitate the formation of π-π stacking. Recently, devices based on BDT units have crossed the efficiency threshold of over 15%, indicating the great potential in the commercialization of this class of material in small-molecule organic solar cells [12,13,14].

**Figure 1:**
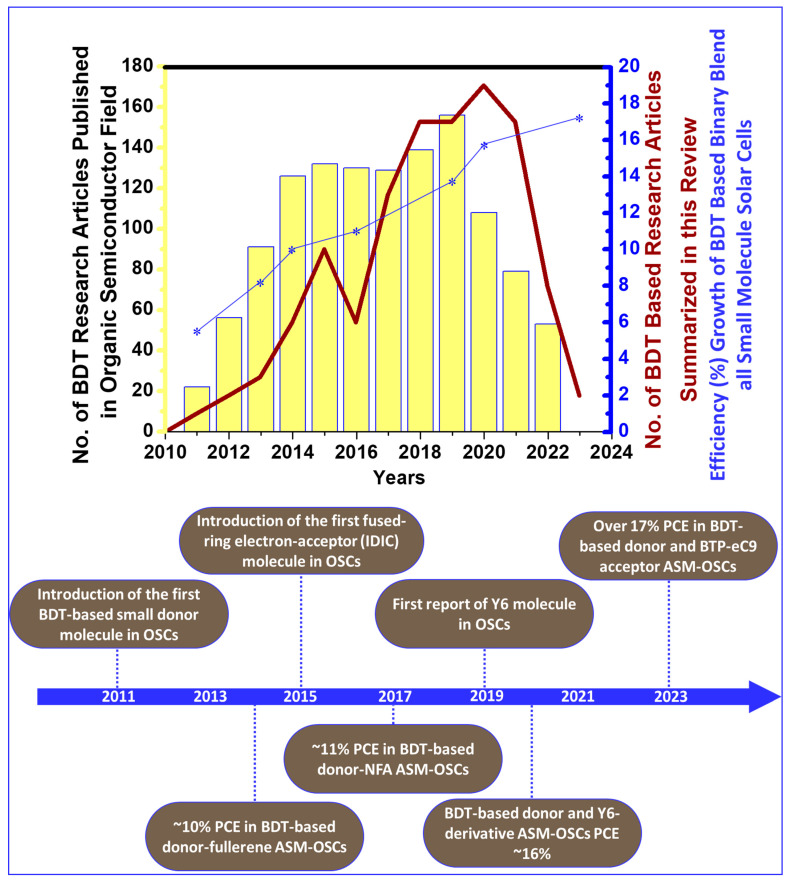
The number of BDT research articles published in the organic semiconductor field (through the keyword “benzo[1,2-b:4,5-b′]dithiophene (BDT)” on Web of Science) and a brief timeline of the development of BDT donor-based ASM OSCs.

The photovoltaic performance of OSC devices with BDT-based small molecules or polymers has kept pace with the development in the field of OPVs. Therefore, summarizing the critical achievements in BDT materials will provide a general guideline for the molecular design strategies of high-performance photovoltaic materials. The number of BDT research articles published in the organic semiconductor field since 2011 (through the keyword “benzo[1,2-b:4,5-b′]dithiophene (BDT)” on the Web of Science) and a brief timeline of the development of BDT donor-based ASM OSCs presented in Figure 1. In this review, we try to summarize the molecular design strategies, chemical structures, and photovoltaic properties of BDT-containing conjugated small molecules developed over the past 10 years. The general synthesis routes of BDT units and their design strategies, including backbone modulation, side chain optimization, end-group engineering, and functional substitutions, are summarized and discussed with several representative examples. Finally, we highlight the problems and challenges facing BHJ-OSCs and provide perspectives for future developments in this exciting research area, which may offer some clues for future BDT material evolutions. We believe that this review will encourage further research on the rational design and synthesis of novel BDT-based organic small molecules with cost-effective, better performance, and high-stability organic solar cells.

## 2. Synthesis and Developments of BDT-Based Donor Molecules in OSCs

**Figure 2:**
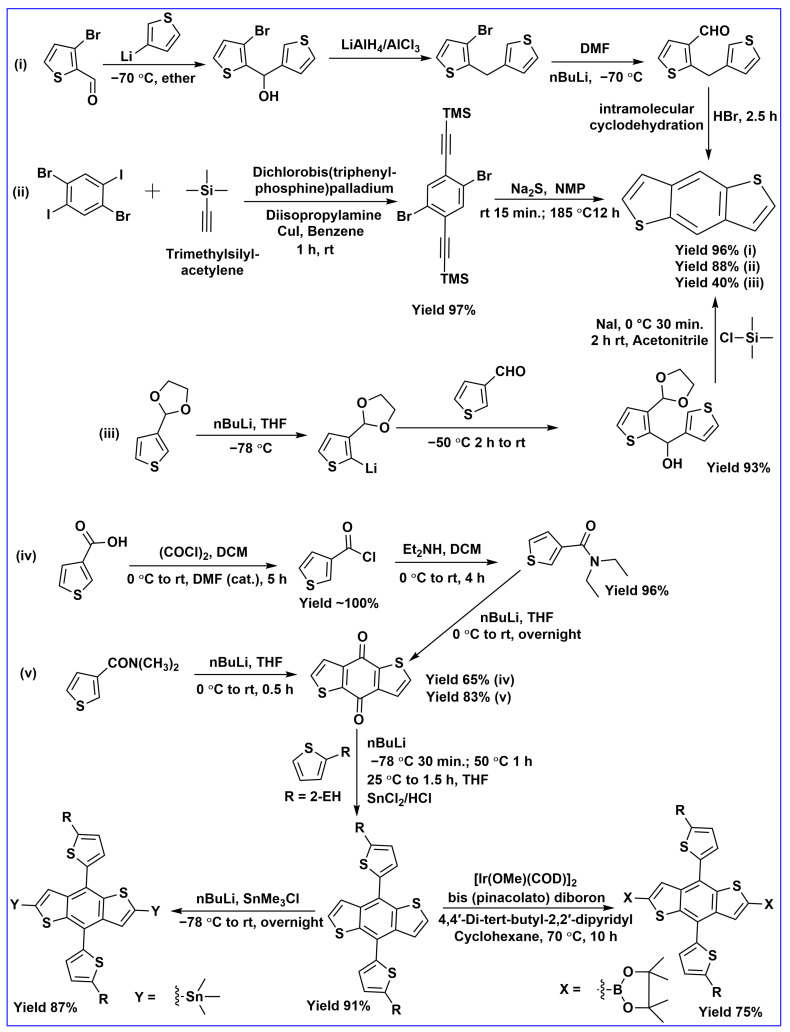
Common synthetic procedures for BDT monomer unit.

In the early 1980s, BDT-based molecules were synthesized and reported for their electrical conducting properties [15]. Subsequently, several organic molecules with BDT units were prepared and applied in organic field-effect transistors (OFETs). Over the years, several synthetic routes were developed in various studies, as shown in Figure 2. Benzannulation engages the construction of BDT from the condensation of two thiophene rings after dehydrative cycloaromatization (Bradsher-type reaction) [16]. The thienannulation reaction shows a more innovative way to construct BDT. More recently, BDT units have been constructed by 3-thiophenecarboxaldehyde (Figure 2) [17]. As shown in Figure 2, the intermediate benzo[1,2-b:4,5- b′]dithiophene-4,8-dione played an important role in the synthesis of a BDT unit with various lateral substitutions to tune the properties of BDT-based molecules. Since Suzuki or Stille coupling are the two most popular reactions involved in the preparation of conjugated BDT organic electronic materials, boronic acid esters or stannane compounds are needed for the reactions with n-butyllithium and then addition of chloro trimethyl stannane or 2 isopropoxy-4,4,5,5-tetramethyl-1,3,2 dioxaborolane [18].

### 2.1. Lateral Side Chain Engineering

**Figure 3:**
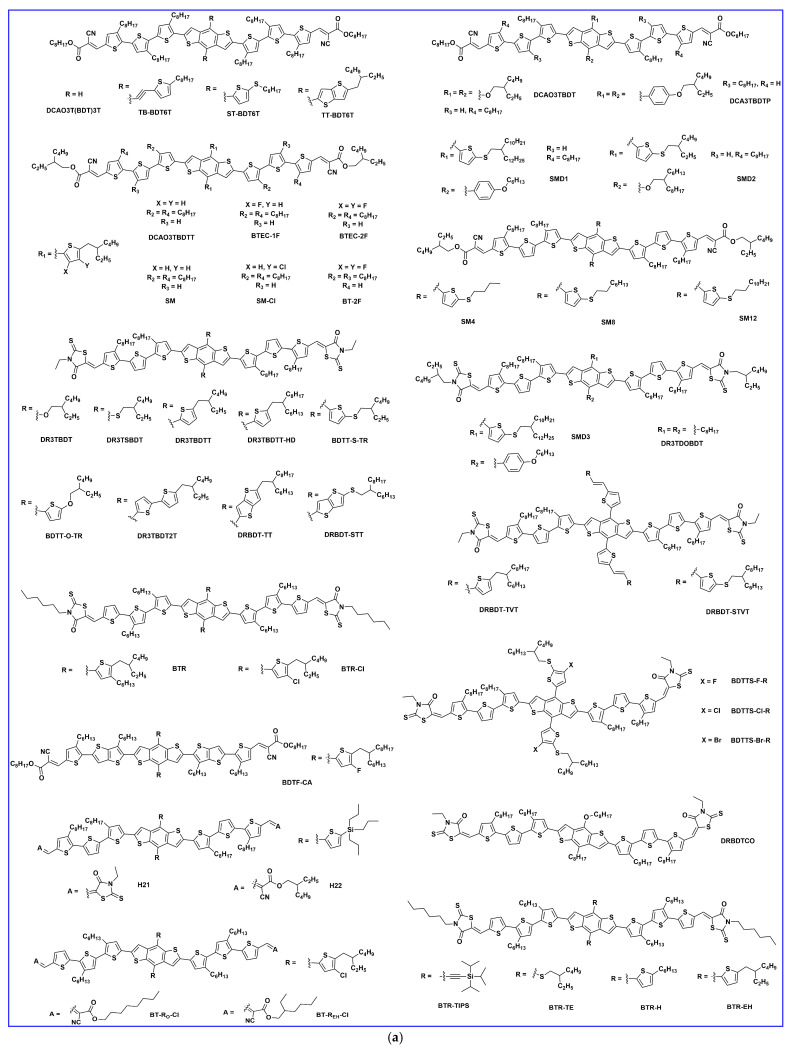

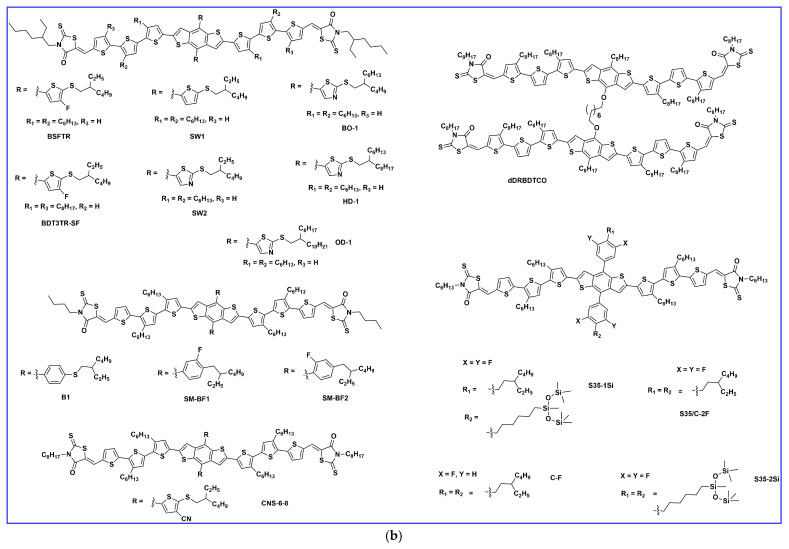
(**a**) BDT molecular structure via lateral side chain engineering. (**b**) BDT molecular structure via lateral side chain engineering.

In 2011, Liu et al. reported the first BDT-based small molecule, DCAO3T(BDT)3T (Figure 3a), by replacing the thiophene unit of the DCAO7T molecule with BDT. The better planar and more electron-rich characteristics of the BDT-based molecule maintained the high FF and increased the photovoltaic performance owing to its increased overall planarity, carrier mobility, and absorption in the solar spectrum. Using simple solution spin-coating, an efficient efficiency of 5.44% (Table 1) was obtained and this work provided a strategic choice for the development of BDT units for solution-processed highly efficient small-molecule OSCs [19]. Patra et al. designed and reported three new small molecules based on different side chains on the BDT central donor unit, named TB-BDT6T, ST-BDT6T, and TT-BDT6T. The optimized BHJ-OSCs devices based on the TT-BDT6T: PC_61_BM (1:0.4, *w*/*w* and 0.05 mg/mL PDMS) blend showed the highest PCE of 5.79%, while the devices based on TB-BDT6T and ST-BDT6T showed a PCE of 3.59% and 4.98%, respectively. The addition of an electron-donating conjugated side chain to the BDT unit changed the optoelectronic properties and performance of the TT-BDT6T solar cell [20]. In 2012, Zhou et al. designed and reported two molecules, DCAO3TBDT and DR3TBDT, with 2-ethylhexoxy substituted BDT as the central building block. The introduction of the 3-ethylrhodanine terminal group in place of the octyl cyanoacetate significantly improved the light absorption and an impressive PCE of 7.38% was obtained in DR3TBDT: PC_71_BM (1:0.8, *w*/*w*) with the addition of 0.2 mg/mL of polydimethysiloxane (PDMS) in the active layer blend [21]. Du et al. synthesized and reported a new two-dimensional (2D) conjugated small molecule, named DCA3TBDTP, with an alkoxyphenyl-substituted BDT unit as the central donor and octyl cyanoacetate as the acceptor unit. The conjugated lateral alkoxylphenyl group was introduced as a weak electron donor, which lowered the HOMO level and resulted in a higher Voc. The BHJ OSC based on DCA3TBDTP: PC_61_BM showed a good efficiency of 4.51% with a high Voc of 0.90 V after thermal annealing at 70 °C [22]. Recently, in 2022, Huang et al. reported three A-π-D-π-A configurations of BDT-based molecules, SMDI, SMD2, and SMD3, by using a symmetry-breaking strategy to achieve ideal phase separation between donor/acceptor by reducing self-aggregation. The 2D aromatic benzene-alkoxyl and thiophene-alkylthiol as lateral side chains on the BDT core and 3-isooctyl rhodanine as the terminal group in SMD3 showed more solar absorption and suitable phase separation properties than SMDI and SMD2, which resulted in the highest PCE of 4.67% among the three when blended with PC_71_BM acceptor [23].

Two novel donors were synthesized and reported as BTEC-1F and BTEC-2F, which were derived from the small molecule DCAO3TBDTT. Enhanced phase separation and carrier transport were attained in BTEC-2F due to more compact molecular packing. Using Y6 as the non-fullerene acceptor (NFA), the device based on difluorinated BTEC-2F achieved a higher efficiency of 13.34% [24]. Later, the same group reported a novel BDT-based donor, BT-2F, which was derived from their previously reported BTEC-2F (by changing the positions and the length of alkyls on terthiophene π-bridges) and blended with NFA acceptor Y6 or N3. The short-length hexyl chains on BT-2F showed more ordered molecular packing and more compact lamellar stacking, and thus improved hole mobility and carrier extraction, which resulted in excellent PCEs of 13.80% and 14.09% for BT-2F:Y6 and BT-2F:N3, respectively [25]. Yongfang Li et al. synthesized and reported a series of new molecules, named SM4, SM8, and SM12, with various lengths of alkylthio lateral side chains to manage the crystallinity and miscibility with the NFA (BO-4Cl). The mid-length alkylthio-substituted donor SM8 achieved a PCE of 13.11% due to more compatible blend miscibility without much loss in charge carrier transport as compared to the shorter and longer lengths of alkylthio contained in SM4 and SM12, respectively [26]. Chen et al. reported two small molecule donors, namely BT-RO-Cl and BT-REH-Cl, with simple terminal alkyl side chain engineering in alkylcyanoacetate to fine-tune the morphology towards high performance. The favorable molecular stacking in blend films with face-on and edge-on combinations with Y6 acceptor provided a fluent 3D transport channel. The terminal octyl chain in BT-RO-Cl showed a PCE of 13.35%, and the branched alkyl side chain showed a better charge transport channel and phase separation in BT-REH-Cl and thus achieved a better PCE of 13.90% [27]. Bin et al. synthesized and reported two new medium-bandgap small molecules, H21 and H22, with an alkylsily-thienyl-conjugated lateral side chain on the BDT central unit. The PCE based on H22: IDIC with the alkylcyanoacetate as the terminal end group achieved 10.29%, which is higher than with 3-ethyl rhodanine as the terminal end group, due to higher charge-carrier mobility and charge pathway in the blend film of H22 [28].

Wu et al. designed a novel BDT-based molecule, BDTF-CA, with a deep HOMO level and fabricated with the narrow bandgap NFA of IDIC with two or four fluorine atoms at the end group acceptor of IDIC. With the increase of fluorine atoms from IDIC to IDIC-4F, the hole and electron mobilities of the active layers increased by one order of magnitude and the device based on BDTF-CA/IDIC-2F gained a PCE of 9.11% [29]. Ge et al. developed a novel small molecular donor, SM-Cl, and the device performance was 7.73% in a binary system with an IDIC acceptor. The ternary device using 10% SM-Cl in SM: IDIC notably increased the PCE to 10.29% (the SM: IDIC PCE is 9.39%) due to the formation of an alloy state which effectively down-shifted the HOMO level of the donor and thus enhanced the Voc [30]. In 2013, Zhou et al. reported three small molecules named DR3TBDTT, DR3TBDTT-HD, and DR3TBD2T. The PCEs achieved 8.12%, 8.02%, and 6.7% for DR3TBDTT, DR3TBD2T, and DR3TBDTT-HD, respectively, with donors: PC_71_BM weight ratio of 1:0.8 *w*/*w* and 0.2 mg/mL of PDMS in the active layer. The better efficiency was attained by improving the short-circuit current density without sacrificing the open circuit voltage and fill factor [31]. Kan et al. synthesized a new molecule based on DR3TBDT with dialkylthiol-substituted BDT as the central building block instead of its dialkyloxy-substituted DR3TBDT counterpart. The new molecule, DR3TSBDT, showed a better photovoltaic property due to the sulfur atom possessing weaker electron-donating ability than the oxygen atom. The optimized device showed an increase in the PCE of 9.95% due to better absorption of blend film and suitable morphology [32]. Cui et al. reported a new small molecule, named BDTT-S-TR, based on an alkylthio-thienyl-conjugated side chain on the BDT central donor unit. The solution-processed BHJ OSC based on BDTT-S-TR: PC_70_BM (1:0.8, *w*/*w*) revealed a high PCE of 9.20% without any extra treatment. Moreover, the device maintained a PCE of ~7.5% up to 300 nm active layer thickness [33]. Later, Min et al. reported the BDTT-based molecules BDTT-TR and BDTT-O-TR. BDTT-based devices with meta-alkoxy side chains exhibited a lower efficiency of 6.50% compared with their meta-alkylthio side chains (7.44%) due to BDTT-TR with alkylthio side chains showing high and well-balanced charge transport properties and suppressed molecular recombination [34]. Kan et al. reported two new molecules based on thieno[3,2-b]thiophene-substituted benzo[1,2-b:4,5-b′] dithiophene, DRBDT-TT, with an alkyl side chain and DRBDT-STT, with an alkylthio side chain. Both molecules showed good thermal stability, ordered molecular packing, and suitable energy levels. The dihedral angle between the thieno [3,2-b]thiophene and BDT moiety was increased after the alkyl chains was replaced by the alkylthio side chain. The best devices based on DRBDT-TT and DRBDT-STT exhibited PCEs of 8.70% and 8.01%, respectively, with a high fill factor over 70% [35]. Li et al. further optimized the PCE of DR3TBDTT with solvent vapor annealing (SVA). Carbon disulfide (CS_2_) SVA led to a larger crystal growth and higher phase purity, which benefited both charge transport and also reduced charge recombination, and thus a higher PCE of 9.58% was obtained [36]. Ni et al. designed and reported the small molecule DR3TDOBDT as having 4,8-dioctyl benzo[1,2-b:4,5-b’] dithiophene as the central block and 3-(2-ethylhexyl)-rhodanine as the end-capped units. The devices showed a low PCE of 4.34% without any post-treatment. After thermal annealing, the efficiency values were improved to 6.53%. When thermal annealing and solvent vapor annealing (TSA) were used, the BHJ device achieved a high PCE of 8.26% with significantly improved fill factor [37].

Chen et al. reported BDT-based core molecules, namely BTR and BTR-Cl (by substituting an alkyl side chain with a chlorine atom on the BTR molecule). The improved crystallinity and matching energy levels of BTR-Cl with Y6 acceptor yielded an efficient PCE of 13.61% [38] Tang et al. reported a highly efficient small molecular donor, BTR-Cl, blended with small molecule acceptor, Y6. The simple concentration-induced morphology demonstrated a high PCE of 14.7% with a Voc of 0.83 V, Jsc of 23.66 mA/cm^2^, and FF of 74.7% [39]. Later, Hu et al. improved the FF in the BTR-Cl: Y6 active layer blend by incorporation of fullerene derivatives. The 5 wt% of PC_71_BM in the active layer blend of BTR-Cl: Y6, fullerene had good miscibility in both donor BTR-Cl as well as acceptor Y6, resulting in reduced bimolecular recombination and thus improved fill factor (77.11%), which demonstrated a record PCE of 15.34% for all-small-molecule organic solar cells [13]. Hou et al. synthesized two small molecules with 1,2-di(thiophen-2-yl) ethane (TVT-substituted) DRBDT-TVT or alkylthio side chains (STVT-substituted) DRBDT-STVT. Both molecules showed a complementary absorption with common acceptors such as PC_71_BM and IDIC. The optimized device based on DRBDT-TVT showed a reasonable efficiency of 6.87% with the PC_71_BM acceptor [40]. A series of molecules, BDTTS-F-R, BDTTS-Cl-R, and BDTTS-Br-R, were synthesized and reported by Ji et al. Higher crystallinity was observed in the BDTTS-F-R molecule due to the non-covalent effect owing to fluorine atoms, while the lower HOMO energy level was observed in BDTTS-Cl-R and BDTTS-Br-R. The suitable energy level, phase separation, and charge carrier mobility in BDTTS-Cl-R attained an efficient PCE of 10.78% [41]. Guo et al. reported two molecules, DRBDTCO and its dimer, dDRBDTCO, with the octamethylene connector. Both molecules showed a high fill factor of 75% and 73%, respectively. The optimized device of DRBDTCO showed a PCE of 8.18%, which is higher than its dimer dDRBDTCO due to the better packing morphology and asymmetrical conjugated side chains [42]. To investigate the structure–property performance relationship, Lee et al. produced four new molecules by side engineering of a BTR-based molecule named BTR-TIPS (triisopropylsilylethynyl), BTR-TE (2-ethylhexylthio), BTR-H (2-hexylthienyl), and BTR- EH (2-(2-ethylhexyl)thienyl). The BTR-TE compound showed an excellent PCE of 9.0% after THF solvent vapor annealing (SVA) for 20 sec. The THF solvent treatment effectively increased the crystallinity of BTR-TE and displayed isotropic ‘rolling log’ orientations of their conjugated backbones with respect to the substrate, which resulted in higher carrier mobility [43]. Later, in 2021, the photovoltaic properties of BTR, BTR-TE, and BTR-TIPS were further increased by blending with Y6 NFA acceptor and in ternary devices. The BTR-TE binary devices showed relatively higher efficiency (13.2%) compared to BTR (11.0%) and BTR-TIPS (8.3%) owing to proper morphology, and increased charge transportation with suppressed recombination. Moreover, the ternary blend device with 10% BTR-TE revealed the best PCE of 16.1% compared with BTR, and BTR-TIPS due to change in nanoscale phase separation upon addition of BTR-TE in the binary blend [44]. By using PC_71_BM and NITI non-fullerene acceptor in ternary device with BTR donor, Zhou et al. suggested synergistically hierarchical morphology which may be reduce the energy loss and enhanced the charge transport through cascade energy level and thus improved the PCE to 13.63% as compared to binary-blend PCEs either with PC_71_BM (9.03%) or NITI acceptor (6.82%) [45]. To address the challenge of morphology in small molecular materials, Yue et al. designed and reported the small donor molecule, BSFTR, and showed that their blend film possessed proper crystallinity, phase separation, and charge transportation, and matched the energy level with Y6 acceptor, resulting in an efficient PCE of 13.69% [46].

To understand the structure–performance relationship with an NFA acceptor, two isomeric acceptors, NBDTP-F_out_ and NBDTP-F_in_, with diverted oxygen position in the benzodi(thienopyran) (BDTP) core were synthesized and fabricated with the molecular donor BDT3TR-SF. The BDT3TRSF: NBDTP-F_out_-blended film obtained a high efficiency of 11.2%, while the BDT3TR-SF: NBDTP-F_in_-blended film showed almost no photovoltaic response (0.02%). The correlation of the interfacial tension between the two combinations showed that proper interfacial tension is a key component for effective phase separation, morphology, and photovoltaic response [47]. Single-bonded thiophene units at the lateral substituted BDT unit have been widely used in OSCs owing to their excellent charge characteristics, but they easily twist and show multiple conformations that are not suitable for enhancement in crystallinity. Qin et al. designed a 2D conjugated small-molecule donor (B1) with a phenyl-substituted BDT central building block, which has a larger rotational barrier, and increased molecular conformation stability, crystallinity, and notably improved PCE compared to BTR with thiophene substitution. B1 is affected and induced from an edge on to a face-on orientation by the acceptor, BO-4Cl, and blend morphology is synergistically optimized. The device based on B1:BO-4Cl obtained an excellent PCE of 15.3% due to its strong interaction with the NFA BO-4Cl in comparison with its corresponding thiophene-substituted BDT-based material, BTR [12]. In 2021, Guo et al. fine-tuned the B1 molecule and reported two new donors, SM-BF1 and SM-BF2, by placing the fluorine atom in the ortho and meta positions. SM-BF1, with an ortho-fluorinated substituent, showed outstanding crystallization properties and better miscibility with Y6 NFA and exhibited more compatible morphology and balanced charge carrier mobilities, leading to a superior PCE of 15.71%, while its analogous SM-BF2 showed a PCE of 10.23% [14]. Recently, siloxane-terminated side chain engineering was used to synthesize three new molecules: S35, S35−1Si, and S35−2Si. All three molecules exhibited dominant edge-on molecular orientation in their neat films, but a huge difference was observed in their blended films with Y6 NFA. The S35−2Si:Y6 blend showed pure face-on orientation, indicating quite different donor: acceptor intermolecular interactions, and obtained an efficient PCE of 13.5% due to more balanced charge transport [48]. In 2022, Lu et al. reported two BDT-based molecules, C-F and C-2F, using side chain engineering with a symmetrically difluorinated benzene ring on the BDT donor core. The OSCs devices fabricated with N3 revealed an efficiency of 14.64% for C-2F: N3 and 7.76% for C-F: N3. The enhancement in the PCE of C-2F:N3 was due to compact molecular packing, increased crystallinity, proper phase separation, and increased and more balanced charge carrier mobility [49]. As is well known, the PCE of BHJ-OSCs is essentially determined by two factors: the active layer materials and their morphology. Recently, a multicomponent system, which introduces a guest component into the host systems in OSCs, has been tested as an effective approach to further increasing the device performance. Feng et al. recently designed a new BDT-based molecule, CNS-6-8, and utilized it in the host PM6: Y6: PC_71_BM system to further tune the morphology of the active layer. Owing to the favorable miscibility of CNS-6-8 in PM6, Y6, and PC_71_BM, the quaternary system achieved good phase separation morphology, enhanced crystallinity, exciton separation, and charge transport carrier, which led to an excellent efficiency of 18.07%, a high fill factor of 78.8%, and a very low voltage loss of 0.54 eV compared to ternary blends (PM6:Y6: PC_71_BM, 0.56 eV) [50]. Two new molecules, SWI and SW2, were designed by incorporating thiophene and thaiazole moieties on the lateral side chain of the BDT core, respectively. Due to the electron-deficient nature of the thiazole unit compared to thiophene, a downshift in HOMO energy level was observed in SW2 which demonstrated an efficient hybridization between charge transfer and local exciton state at the D/A interface, driving a larger Voc compared to SW1, and provided an effective PCE of 15.51% [51]. Later, in 2023, the same group reported three molecules, BO-1, HD-1, and OD-1, with alkylated thiazole side groups which differ only in the alkyl side chain length on the thiazole unit. The length of alkyl side chain significantly influences the crystallinity and blend morphology of the active layer. Owing to decent BHJ morphology, the HD-1 based molecule achieved a champion device efficiency of 17.19% with the BTP-eC9 acceptor, which is currently one of the highest PCEs among SM-OSCs [52].

### 2.2. End Group Engineering

**Figure 4:**
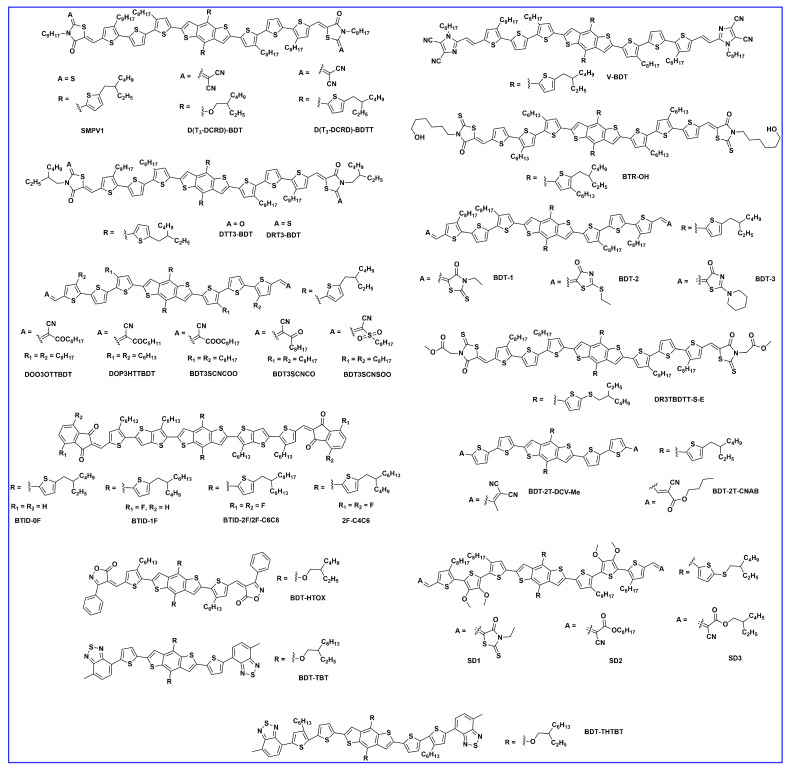
BDT molecular structure via end group engineering.

Liu et al. synthesized and reported a 2D conjugated organic small molecule named SMPV1 (Figure 4). The fabricated device with a PC_71_BM acceptor revealed a PCE of 7.2%. After the addition of 0.5 mg/mL, the device efficiency improved to 8.1% (Table 2) due to nanoscale phase separation and bicontinuous interpenetrating network [53]. Fan et al. designed and reported two new small molecular donors, D(T3-DCRD)-BDT and D(T3-DCRD)-BDTT. These molecules are based on 2-(1,1-dicyanomethylene) rhodanine (DCRD) as an electron withdrawing end-group into BDT- and BDTT-central donor units, respectively. The efficiency of 1.10% for D (T3-DCRD)-BDT and 1.94% for D (T3-DCRD)-BDTT was achieved with the PC_61_BM acceptor. The enhanced performance of the latter molecule is due to its broader absorption and higher hole mobility due to the introduction of conjugated thiophene side chains [54]. Kumar et al. reported two new molecules, DRT3-BDT and DTT3-BDT, constructed with rhodamine-based different acceptor units. The performance of the DRT3BDT and DTT3-BDT achieved 6.76% and 5.25%, respectively, after optimization with 3% (*v*/*v*) DIO. The better nanoscale phase separation, higher carrier mobility, and smooth surface in the DRT3BDT: PC_71_BM-blend film were responsible for better efficiency compared to the DTT3-BDT-based device [55]. Deng et al. designed and reported two new small molecules: DOO3OTTBDT and DOP3HTTBDT (by shortening alkyl chains that are attached to π-conjugated bridge end-capped acceptors). The effective molecular packing and higher crystallinity with the addition of 0.25% (*v*/*v* DIO) in donors: PC_71_BM blend achieved a high PCE of 5.64% and 5.26% for DOP3HTTBDT and DOO3OTTBDT, respectively [56]. Similarly, the same group reported two new molecules, BDT3SCNCOO and BDT3SCNSOO, using subtle changes in the molecular structure of their previous work. The fabricated devices revealed a PCE of 6.4%, 6.4%, and 3.0% for BDT3SCNCOO, BDT3SCNCO, and BDT3SCNSOO, respectively.

The PCE of the BDT3SCNCOO-based device is relatively higher due to its shorter stacking distance and small steric effect, resulting in an effective FF of 72%, whereas the PCE of the BDT3SCNSOO-based device is lower due to poor crystallinity and FF [57]. Deng et al. reported three small molecules, named BTID-0F, BTID-1F, and BTID-2F, that contained gradient-electron density end acceptors substituted with various fluorine atoms. The efficiencies for the inverted devices were 8.30%, 10.4%, and 11.3% for BTID-0F, BTID-1F, and BTID-2F, respectively, with the PC_71_BM acceptor. Fluorination leads to an optimal active layer morphology, enhanced domain purity, the formation of hierarchical domain sizes, and a directional vertical phase separation. The optimal morphology balances charge separation and transfer and thus facilitates charge collection. As a consequence, fluorinated molecules exhibited excellent inverted device performance with a PCE of 11.3% for a two-fluorine atom substituted molecule, which was the highest achieved efficiency for small molecule OSCs at that time [58]. Similarly, the same group reported two molecules, 2F-C4C6 and 2F-C6C8, and the OSC devices with IDIC acceptor revealed an efficiency of 6.41% and 8.23%, respectively. The AFM images showed relatively higher crystallinity in the 2F-C4C6 donor owing to its smaller alkyl side chains and thus earlier crystallization, which ultimately led to poor performance [59]. A new molecule named V-BDT in the A-π-D-π-A configuration, based on a vinazene end group acceptor, was reported by Chen et al., and the device obtained a PCE of 3.73% after thermal annealing for 10 min. at 75 °C and then chloroform vapor annealing for 40 s [60]. To fine-tune the morphology of the host binary blend, optimize the morphology, enhance the absorption, and improve charge transport, BTR-OH was used in ASM OSC as a ternary component in BTR: PC_71_BM. BTR-OH: PC_71_BM binary blend showed an inferior PCE of 8.00% with weaker crystallinity and phase separation, while after adding BTR-OH, the BTR: PC_71_BM binary blend exhibited a uniform and smooth film with suitable crystallinity and yielded an improved PCE of 10.14% with an active layer thickness of ~300 nm [61]. Che et al. reported a series of BDT-based molecules, BDT-1, BDT-2, and BDT-3, with different rhodanin-derived end groups. Among the three, the BDT-1 with an N-alkylthiazolonethione end group showed the highest efficiency of 5.46% with a fullerene acceptor due to its strongest electron-withdrawing ability and lowest bandgap. The PCE of BDT-2 and BDT-3 dropped to 2.99% and 0.38%, respectively, because of a decrease in the hole mobilities, which hampered the charge extraction and thus reduced the Voc, Jsc, and FF [62]. Chang et al. synthesized a new medium-bandgap BDT-based donor molecule, DR3TBDTT-S-E, and incorporated it into two types of ternary solar cells with two donors and one acceptor (PC_71_BM/IDIC). The DR3TBDTT-S-E molecule in the binary DR3TBDTT: PC_71_BM host blend lead to an enhanced PCE from 9.09% to 10.38 owing to suppressed charge recombination and improved charge transportation and charge extraction. The DR3TBDTT-S-E donor molecule in the DCAO3TBDTT: IDIC host blend showed a mixed face-on and edge-on orientation, creating 3D charge pathways that assisted the charge transportation and thus increased the PCE from 9.49% to 10.04% [63].

Guo et al. synthesized two novel BDT-based molecules using two different end group acceptors, dicyanovinyl and n-butyl cyanoacetate, for BDT-2T-DCV-Me and BDT-2T-CNAB, respectively. The photovoltaic performance based on the BDT-2T-CNAB: IDIC blend exhibited higher Jsc and fill factor, and thus much higher PCE of 6.17% compared to those of BDT-2T-DCV-Me: IDIC devices (1.56%). In comparison to the BDT-2T-DCV-Me system, the BDT-2T-CNAB-based device showed smoother film surface morphology, superior exciton dissociation, charge generation, and charge carrier mobilities, as well as lower non-geminate recombination losses [64]. To investigate the effect of the combination between a strong donor and strong acceptor, a new BDT-based donor molecule, BDT-HTOX was synthesized by Sylvianti et al. using phenylisoxazol (OX) as an acceptor. In the inverted configuration using the PC_71_BM acceptor, the PCE obtained 0.80% [65]. Sylvianti et al. reported BDT-based molecules, named BDT-TBT and BDT-THTBT, using benzothidiazole as the acceptor moiety. The BDT-THTBT showed broad absorption and decreased bandgap owing to extended conjugation length compared to the BDT-TBT and resulted in a PCE of 1.04% [66]. Duan et al. developed new BDT-based molecules, named SD1, SD2 and SD3, by combining the modification in the side chains in the π-bridge with different end group acceptors. The introduction of alkoxy group in thiophene π-bridge induced intermolecular S…S and intramolecular S…O non-covalent interactions for higher planarity of these small molecules. The SD1:Y6-T blend showed more favorable face-to-face packing and ideal morphology for efficient charge dissociation and transport, which contributed to a higher PCE of 10.12%. From SD1: Y6-T to SD3: Y6-T, the face-to-edge packing was increased, which resulted in lowered exciton dissociation efficiency and consequently decreased the device efficiencies from 8.28% to 5.79% for SD2: Y6-T to SD3: Y6-T, respectively [67].

### 2.3. π-. Bridge Engineering

**Figure 5:**
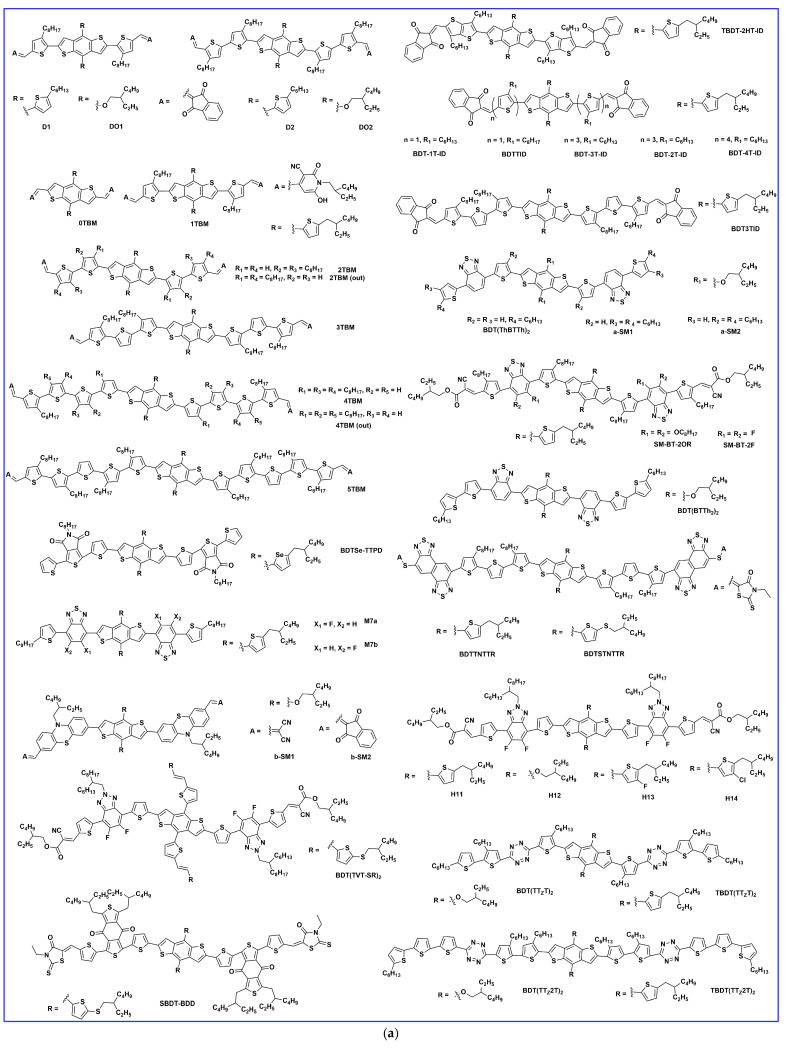

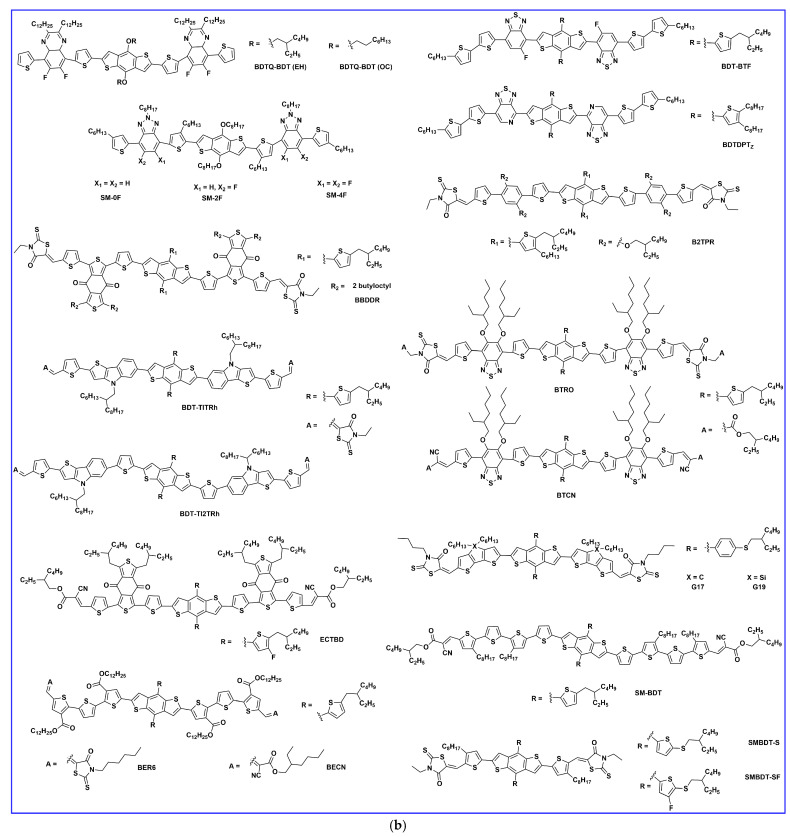
(**a**) BDT molecular structure via π-bridge engineering. (**b**) BDT molecular structure via π-bridge engineering.

Shen et al. designed and reported four small donor molecules: D1 and D2, with hexylthienyl, and DO1 and DO2, with 2-ethylhexyloxy side chains, on the BDT unit (Figure 5a). The PCEs of the OSCs based on the donors and PC_70_BM acceptor were 6.75%, 5.67%, 5.11%, and 4.15% (Table 3) for D2, D1, DO2, and DO2, respectively. D2 and DO2, with bithiophene π-bridges, exhibited stronger absorbance and higher hole mobilities than the compounds with only thiophene π-bridges in D1, DO1. Additionally, this result demonstrated that the side chain with thienyl group on the BDT unit was better than that of the alkoxy side chain [68]. Tang et al. synthesized and reported a series of small molecules based on BDTT as the central donor and electron-deficient quinoidal methyldioxocyano-pyridine (MDP) by targeting oligothiophene (0T–5T) π-bridges on its various positions. The crystallinity, fibril length, phase size of the blend films, and performance of devices were finely tuned by increasing the size of the oligothiophene from 0 to 5 thienyl units, as well as the alkyl chains on the thiophene bridge from “outward” to “inward”. With the increase of the oligothiophene bridge, induced intramolecular charge transfer occurred, leading to the absorbance enhancement. The higher and more balanced hole and electron mobilities for the 3TBM led to the highest PCE of 6.29% among them [69]. Deng et al. reported a new molecule TBDT-2HT-ID by subtle modification of their previous BTID-0F molecular structure. The good crystallinity, edge-on molecular packing, and high hole mobility resulted in an efficient PCE of 7.4% with the PC_71_BM acceptor without any post-treatment [70]. Komiyama et al. designed and synthesized a series of BDT-based A−π−D−π−A small molecules, BDT-nT-ID (*n* = 1–4), using 1,3-indandione (ID) as the terminal electron-accepting (A) units. The effect of the length of the thiophene π-bridge units on morphology, mobility, and photovoltaic performance were systematically investigated. Among these molecules, the highest PCE was obtained for BDT-2T-ID at 6.9% due to low series resistance and effective exciton dissociation at BHJ [71]. Similarly, Lee et al. designed and reported two novel small molecules, named BDTTID and BDT3TID. The PCEs of optimized devices for BDTTID and BDT3TID blends with PC_70_BM acceptor achieved 5.54% and 4.74%, respectively. Furthermore, the BDTTID-based device showed superior thermal stability upon thermal treatment at 100 °C for 40 h. The PCE of the BDTTID-based device remained above 66% of its initial PCE, whereas the PCE of the BDT3TID-based device was dramatically reduced from 4.60 to 1.11%. These results revealed the importance of thermal annealing temperature and controlling the crystallinity for efficient and stable OSC devices [72]. To study the impact of moiety order in the chemical structure of small molecules and their photovoltaic responses, two isomeric compounds, namely, BDT(ThBTTh)_2_ and BDT(BTTh_2_)_2_, were reported by Liang et al. In comparison with the isomer BDT(BTTh_2_)_2_, BDT(ThBTTh)_2_ possesssing a slightly structural deviation in exchange of BT and thiophene positions showed a lower melting point, a blue-shifted absorption spectrum in solution, and a slightly low-lying HOMO. More importantly, BDT(ThBTTh)_2_ exhibited a significantly ordered crystalline structure, especially in the blend film with PC_61_BM acceptor, having a considerably larger hole mobility and resulting in a PCE of 4.53% for BDT(ThBTTh)_2_ compared to 1.58% for BDT(BTTh_2_)_2_ [73]. Sufficient solubility of organic materials is highly desirable for solution processability and effective performance. To address the poor solubility issue, Yao et al. reported a pair of isomers, a-SM1 and a-SM2, based on their previously reported molecule (BDT(ThBTTh)_2_). The two additional hexyl side chains in the inner thiophene units provided a better twisting conjugated backbone in a-SM2 than containing two additional hexyl side chains at the terminal thiophene units, resulting in tighter molecular packing, better morphology, larger hole mobility, and thus leading to an efficiency of 2.57% for a-SM2 compared to 1.40% for a-SM1 [74].

To investigate the effect of the fluorine substitution and their OSC performance, Qiu et al. reported two BDT-based donors: SM-BT-2OR with an alkoxy side chain and SM-BT-2F with a fluorine atom substitution at the benzothiadiazole (BT) acceptor. The as-cast OSC devices with IDIC acceptor showed an efficiency of 2.33% and 2.76% for SMBT-2OR and SM-BT-2F, respectively. Interestingly, when thermal annealing (120 °C for 10 min) was applied on both devices, the SMBT-2OR revealed an increased PCE of 7.20%, while the SM-BT-2F displayed an even lower PCE of 1.60%, which was ascribed to the large phase separation and resulted in a decrease in the exciton dissociation and charge transportation after TA treatment [75]. Kim et al. reported a BDT-based molecule, BDTSe-TTPD, that contained the selenophene lateral side chains on the central BDT unit. The selenophene moiety induced the inter-chain interactions between BDTSe-TTPD chains due to strong Se–Se interactions, and the incorporation of the Se building block lowered the HOMO energy level of the BDTSe-TTPD compared to the corresponding thiophene building block. The optimized device with PC_71_BM acceptor showed a reasonable efficiency of 4.37% [76]. To investigate the effect of fine-tuning molecular properties and related photovoltaic performance, Duan et al. designed and reported two BDT-based isomers, M7a and M7b, with F-atom position (proximally and distally toward the BDT central unit). The effect of the F-atoms’ positions indicated considerable dissimilarities in their thermal, optical, and electrochemical properties. In M7a, F atoms at the proximal position indicated broader absorption and better photovoltaic performance, leading to an efficiency 3.9% higher than in M7b (F-atoms at distal positions PCE of 2.5%) [77]. Two new molecules, b-SM1 and b-SM2, were synthesized with the BDT core linked with a phenothiazine (PTZ) π (donor) unit and 1,3–indanedione and malononitrile end group units, respectively. After the optimization of nanoscale morphology using carbon disulphide (CS_2_) SVA treatment of the blend film, the resultant OSCs revealed a significant PCE of 6.20% and 7.45% for b-SM1 and b-SM2, respectively [78]. Guo et al. reported a novel alkylthio-thienylenevinylene thiophene (TVT-SR) containing a lateral side chain on the central BDT unit, named BDT(TVT-SR)_2_. The device based on BDT(TVT-SR)_2_ with an IDIC acceptor revealed a significant PCE of 11.1% after thermal annealing due to improvements in film morphology, appropriate phase separation, and charge transport properties [79]. A novel medium bandgap BDT-based small molecule, SBDT-BDD, consisting of two electron accepting units, rhodanines and benzo-[1,2-c:4,5-c′]dithiophene-4,8-diones (BDD) was designed in an A1-A2-D-A2-A1-type configuration. The dual electron-accepting units provided the SBDT-BDD with complementary absorption and a suitable energy level with the fused-ring electron acceptor IDIC, leading to an efficient PCE of 9.2%. Moreover, the SBDT-BDD was further investigated in ternary devices using the PC_71_BM acceptor and showed an outstanding efficiency of 10.9% owing to ideal film morphology, efficient charge separation, and suppressed charge recombination [80]. Similarly, Wan et al. synthesized two novel molecules, BDTTNTTR and BDTSTNTTR, by using naphtho[1,2-c:5,6-c]bis[1,2,5]thiadiazole (NT) as the second internal acceptor, which displayed π-bridge with terthiophenes on both sides and 3-ethylrhodanine end-group acceptor unit. Owing to extended conjugation length and strong electron affinity, both NT-based materials showed high crystallinity, larger hole mobilities, and low HOMO levels. When a halogen-free solvent (CS_2_) was used, BDTSTNTTR containing sulfur atoms in lateral side-chain devices, showed a greater Voc of 0.93 V and an efficient PCE of 11.53%, with a small voltage loss of 0.57 eV compared to the 10.02% PCE for BDTTNTTR [81]. Bin et al. designed and reported two novel 2D BDT and 1D BDT donor molecules, H11 and H12, respectively. To extend the conjugation length, absorption profile, and electron-accepting profile, two electron acceptor units, fluorobenzotriazole as the internal acceptor between thiophene on both sides and alkyl cyanoacetate as the end-group acceptor, were incorporated into both molecules. The 2D-conjugated H11 showed stronger absorption, lower-lying HOMO energy level, higher hole mobility, and more ordered bimodal crystallite packing than H12 (with alkoxyls) led to a PCE of 9.73% for H11: IDIC compared to 5.51% for H12: IDIC [82]. Based on their previous work, the same group reported two new molecular donors, H13 and H14, fine-tuned by fluorine and chlorine substitution of the original donor molecule H11, respectively. The chlorinated H14 molecule exhibited a higher degree of crystallinity, smaller π-π distortion, and nanomorphology, which enabled the authors to generate and collect the charge more effectively, and improved open-circuit voltage, thus showing an efficient PCE of 12.1% with IDIC-4F acceptor compared to 10.3% for H13 [83].

Ma et al. reported two new BDT-based donor molecules BDT(TTzT)_2_ and BDT(TTz2T)_2_ in a D2-A-D1-A-D2-type configuration using tetrazine(Tz) as an electron acceptor end-group, and bithiophene or terthiophene as the end donor units. The introduction of the tetrazine acceptor unit effectively reduced the HOMO energy level of both molecules, and after the optimization with DIO, the device showed an efficiency of 5.01% and 5.29% for BDT(TTzT)_2_ and BDT(TTz2T)_2_, respectively [84]. Similarly, the same group reported two new molecules, named TBDT(TTzT)_2_ and TBDT(TTz2T)_2_, by increasing the number of thiophene units. The increased thiophene units extended the conjugation length, upshifted the HOMO energy level, and reduced the bandgap, which led to a reasonable efficiency of 6.10% and 6.56% for TBDT(TTzT)_2_ and TBDT(TTz2T)_2_ [85]. Park et al. reported two new molecules, BDTQBDT(EH) and BDTQ-BDT(OC), using 2,3-didodecyl-6,7-difluoro-5,8-di(thiophen-2-yl) quinoxaline (DTQ) as the electron acceptor unit. The OSCs devices based on BDTQBDT(EH) and BDTQ-BDT(OC) showed an efficiency of 1.20% and 0.83%, respectively. These results demonstrated that the fine alteration in the lateral alkyl side chains exhibited a considerable change in the thermal, optical, energy levels, and photovoltaic properties of both molecules [86]. To investigate the effect of fluorination on the photophysical properties of small molecules, Gu et al. designed and reported three new molecules, SM-0F, SM-2F, and SM-4F, using benzotriazole (BTA) or fluorinated BTA. The fluorination on the BTA unit lowered the HOMO energy levels and possessed higher electron mobility than SM-0F. The optimized devices showed a PCE of 2.56%, 3.94%, and 3.48% for SM-0F, SM-2F, and SM-4F, respectively [87]. Guo et al. reported a new A-π-D-π-A0type molecule, BBDDR, based on the central BDT donor core, benzo[1,2-c:4,5-c’]dithiophene-4,8-dione (BDD), together with two thiophene units as the π-bridge, and rhodanine as the electron-accepting end group. The optimized device based on BBDDR: IDIC with chloroform solvent vapor annealing (CF-SVA) treatment exhibited a PCE of 7.8% and a high Voc of 1.01 V, owing to higher and more balanced charge carrier mobilities than the as-cast device [88]. Huang et al. reported two new A-π-D-π-A-type small molecules, BDT-TITRh and BDT-TI2TRh, which contained 2-(thiophen-2-yl)-N-alkyl-thieno [3,2-b]indole (TIT) or 2,6-di(thiophen-2-yl)-N-alkyl-thieno [3,2-b]indole (TI2T) as π-bridge and 3-alkyl-rodanine as the electron-accepting unit. BDT-TI2TRh with TI2T (extended π-bridge) showed a stronger absorption and higher hole mobility, and resulted in a PCE of 4.19%, while the BDT-TITRh revealed a lower PCE of 3.52% [89]. A new planar D2-A-D1-A-D2-type structure, BDT-BTF, containing fluorinated benzothiadiazole (BTF) as an electron acceptor unit and hexyl dithiophene as an end group, was reported by Wang et al. and used in OSCs with the PC_71_BM acceptor. Benefiting from its strong crystallinity and coplanarity, BDT-BTF showed a high hole mobility in blend film and revealed an efficiency of 5.88% with a very low content of PC_71_BM acceptor [90]. Similarly, the same group modified their previously reported BDT-BTF molecule by replacing BTF with a [1,2,5]thiadiazolo[3,4-c]pyridine (PTz) acceptor moiety to enhance the electron-withdrawing ability and film morphology. The new BDTDPTz molecule showed better absorption, lower HOMO energy level, better film quality, and strong crystallinity compared to BDT-BTF and thus revealed a PCE of 6.28% with the PC_71_BM acceptor [91]. Wang et al. synthesized a BDT-based new donor, B2TPR, by using thiophene-(p-ethylhexyloxy benzene)-thiophene as the π-bridge and methyl rhodanine as an acceptor. The intramolecular rigidity and planarity of B2TPR increased through non-covalent interaction, and a high and balanced charge carrier mobility was obtained, which reduced the bimolecular recombination, and the resulting optimized device obtained a reasonable PCE of 7.10% [92]. Meng et al. designed and reported two new molecules, BTRO (through incorporation of rhodamine and 2-alkyl acetate together as an end-group) and BTCN (using alkyl cyanoacetate acceptor only). In comparison to BTRO, BTCN had a wider bandgap; however, BTCN exhibited a higher and balanced carrier mobility, a higher extinction coefficient, and better complementary light absorption with the IDIC-4F acceptor, and thus led to an efficiency of 4.08% [93]. Ternary OSCs have received much attention for improving the PCE through complementary absorption and morphological compatibility of blend film. A new BDT-based donor, ECTBD, was developed and used as a third component in the PM6:Y6 blend and revealed an efficient efficiency of 16.51%, which was much higher than ECTBD: Y6 (1.58%). The cascade distribution of energy levels between ECTBD, PM6, and Y6 provided sufficient driving force for charge transfer [94]. In 2021, two new BDT-based A−π−D−π−A configurations, BER6 and BECN, consisting of diester-terthiophene as a π-bridged unit and alkyl rhodanine or alkyl cyanoacetate as a terminal electron-acceptor unit, were designed and reported. The BER6/IDIC-based devices displayed an efficient PCE of 9.03%, while the devices based on BECN/IDIC displayed a much lower PCE of 5.52% owing to unevenly distributed microcrystalline particles on the surface of BECN/IDIC [95]. Two new small molecules with a central BDT donor, G17 and G19, using cyclopentadithiophene (CPDT) and dithienosilole (DTS) as the π donor moieties, respectively, were synthesized and reported. Both the SMs utilized in a ternary system and the device based on D18-Cl:G17 (0.9:0.1 *w*/*w*):Y6 and D18-Cl:G19 (0.9:0.1 *w*/*w*):Y6 achieved a record efficiency of 17.13% and 18.53%, respectively. A carbon π-bridge containing sp3 hybridization showed an amorphous orientation, while the silicon-substituted G19 showed highly ordered, suitable phase separation in the ternary blend, better charge transfer, and extremely edge-on orientation, causing the efficiency of G19 [96]. Li et al. reported two novel molecules: SM-BDT using a BDT central donor and SM-DTBDT using dithieno benzodithiophene (DTBDT) central donor core unit. The devices optimized with a Y8 acceptor revealed a reasonable PCE of 12.45% and 10.68% for SM-BDT and SM-DTBDT, respectively. Both molecules showed good film morphology, efficient exciton dissociation, and charge transportation [97]. Lee et al. developed two new BDT-based molecules, SMBDT-S and SMBDT-SF, with octyl thiophene π-bridges and a rhodamine end-group. Fluorination in the lateral side chain of the SMBDT-SF molecule effectively reduced the HOMO level (−5.72 eV as compared to SMBDT-S, −5.56 eV), which resulted in a significantly high Voc of 1.18 eV. The optimized devices of SMBDT-S and SMBDT-SF showed an efficiency of 2.9% and 0.9%, respectively. The relatively low PCE of both molecules was due to low photocurrent, especially in SMBDT-SF (1.4 mA/cm^2^), poor film morphology with large aggregates, and the difficult solubility of both molecules [98].

### 2.4. Miscellaneous BDT-Based Small Donor Molecules

**Figure 6:**
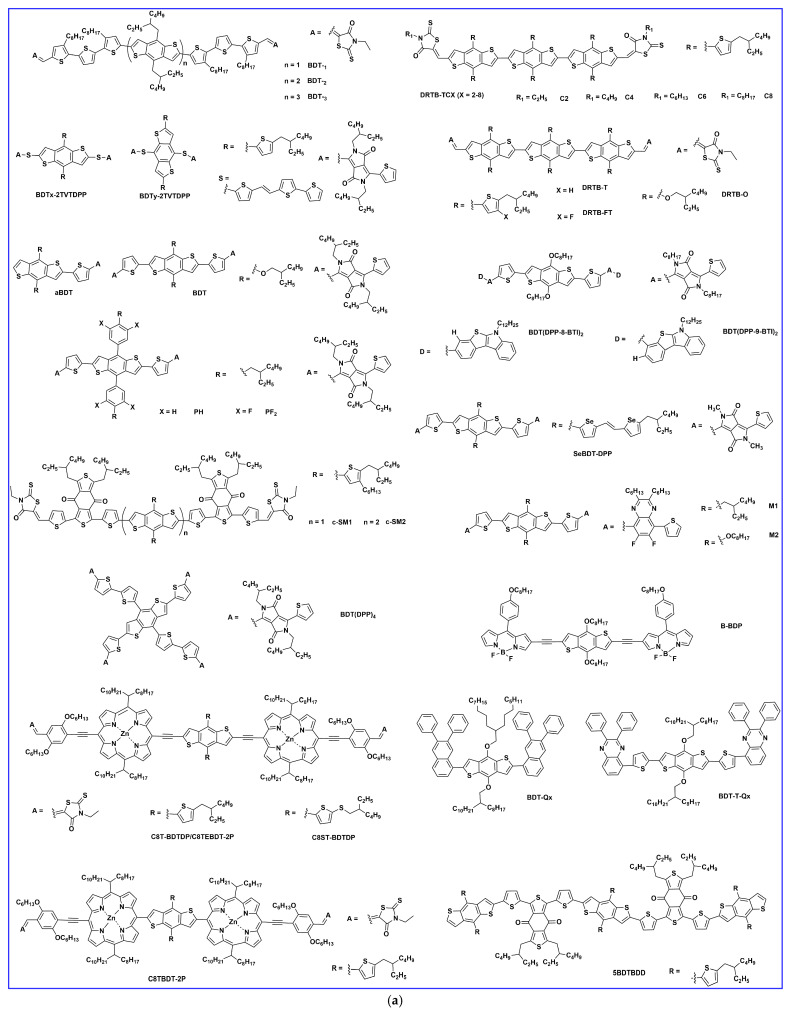

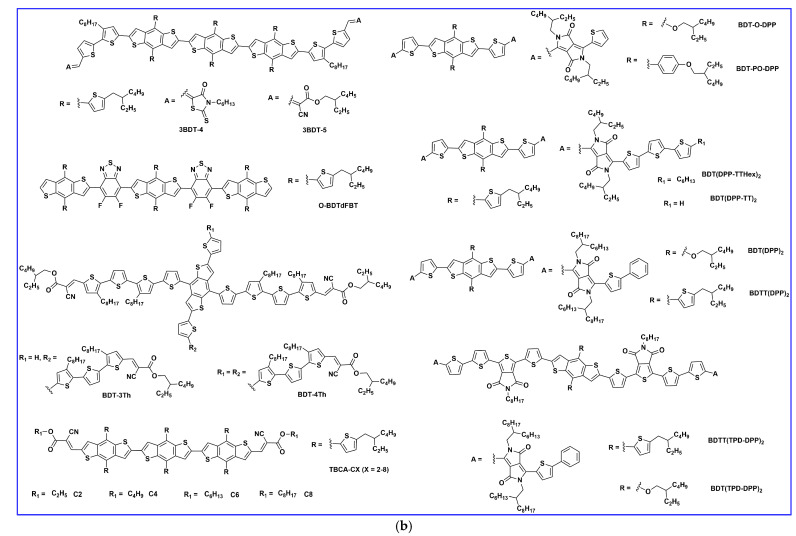
(**a**) BDT molecular structures with various combinations. (**b**) BDT molecular structures with various combinations.

**Figure 7:**
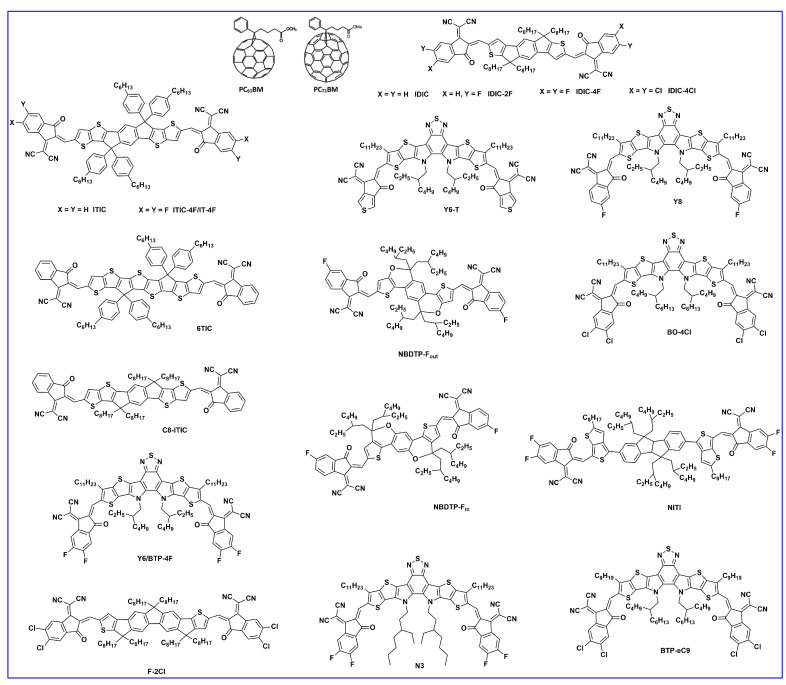
Chemical structures of the small molecular acceptor address in this review.

To investigate the effects of the number of BDT central donors and their intermolecular interaction between BDT units, bulk morphology, exciton diffusion, and charge transport properties, Lee et al. first demonstrated a series of BDT-based small-molecule donors (BDT1, BDT2, and BDT3) bearing 1–3 BDT units (Figure 6a). In comparison to BDT-_1_, BDT-_2_ and BDT-_3_ showed the existence of strong intermolecular interaction between BDT units. Moreover, the BDT2 thermogram indicated co-existence of the two clear crystalline phases, and such strong intermolecular interactions in BDT-_2_ produced the suitable interpenetrating network in the BHJ film, which enhanced exciton diffusion and charge transport. Consequently, BDT-_2_ revealed an efficiency of 8.56% and a 7.45% (Table 4) efficiency in a rigid module of 77.8 cm^2^ [99]. Duan et al. designed and reported two isomeric BDT-based molecules, BDTx-2TVTDPP and BDTy-2TVTDPP, by using thienylenevinylene-thiophene (TVT) and diketopyrrolopyrrole (DPP) as a π-bridge and acceptor, respectively. The BDTy-2TVTDPP-based devices showed better PCE (2.85%) than the BDTx-2TVTDPP (1.58%) owing to extended absorption, better solubility, deep HOMO level, and smoother blend film morphology than BDTx-2TVTDPP [100]. The effects of molecular shape and their intermolecular connection and bulk film morphology were investigated by designing a series of molecules, aBDT, BDT, BDF, NDT, and zNDT, using one or two electron-deficient diketopyrrolopyrrole (DPP) moieties. The one-sided DPP acceptor small molecule (Figure 7), aBDT, resulted in low conjugation length, increased π–π length, a high reorganization energy, and low hole mobility, and thus resulted in a poor efficiency of 0.4%, whereas the addition of a second DPP unit in the BDT increased the molecular planarity, decreased reorganization energy, and improved hole mobility over aBDT and showed a reasonable PCE of 3.6% [101]. A series of molecules with BODIPY linked through several donor units, such as fluorene, carbazole, benzodithiophene, and phenothiazine, namely F-BDP, C-BDP, BBDP, and P-BDP, respectively, were designed and reported. Among them, the BDT-based B-BDP molecule revealed the highest PCE of 4.65% due to its short non-covalent S….S interaction with adjacent molecules, strong red shift in solid state film, and favorable BHJ morphology [102]. Mark et al. reported two new BDT-based molecules, PH and PF2, using diketopyrrolopyrrole (DPP) as an electron acceptor group but with or without fluorination in lateral BDT side chains. Ternary OSCs with PC_61_BM acceptor showed that an electronical alloy formed after mixing with both donors and increased the PCE from the optimized binary blend (PF2: PC_61_BM) 4.26% to 4.90% (PH:PF2: PC_61_BM) [103]. The longer chains are often used to enhance solubility and film quality. Using this strategy, Yang et al. synthesized a series of alkylthienyl-substituted BDT trimers, named DRTB-T-CX, which differed in acceptor end-group alkyl chains. Extension of alkyl length showed variation in molecular orientation, and thus DRTB-T-C2 exhibited an edge-on molecular orientation with lamellar packing diffraction in the out-of-plane (OOP) direction and π–π stacking diffraction in the in-plane (IP) direction, while DRTB-T-C4 showed a better face-on orientation with π–π stacking mostly in the OOP direction. The face-on orientation was proficient at enhancing the π–π stacking and charge mobilities; thus, the DRTB-T-C4 with IT-4F acceptor film revealed an efficiency of 11.24% and retained a sufficient PCE of 10% even at a high active layer thickness of 300 nm [104]. To enhance the planarity and transfer from 1D to 2D BDT-based molecules, the substitution in the 4 and 8 positions of BDT by alkylthienyls has been widely approached. Utilizing this idea, Hou et al. reported two BDT-based donors: DRTB-O and DRTB-T. The OSC devices based on DRTB-O: PC_71_BM and DRTB-T: PC_71_BM exhibited PCEs of 7.08% and 4.91%, respectively. Moreover, along with the IDIC acceptor, the difference in efficiency observed was very high, and DRTB-T showed a remarkably higher PCE of 9.06% compared to DRTB-O (0.15%). The poor efficiency in the DRTB-O system was due to large aggregation and strong phase separation between the donor and acceptor, which further prevented the formation of a charge transport channel. Additionally, the high-polar-alkoxyl-function group tended to form strong interactions with the end groups in IDIC, so the intermolecular interaction between DRTB-O and IDIC could be quite strong and the large granular aggregations formed hindered the charge transportation in the DRTB-O: IDIC system [105]. Similarly, a new molecule, DRTB-FT, was synthesized using fluorine atoms on the thienyl position of the lateral BDT side chin. By blending with the NFA acceptor, F-2Cl, the optimized device gave a reasonable PCE of 7.66% with a high Voc of 1.07 V [106]. Qi et al. reported a novel type of BDT molecules, BDT(DPP-8-BTI)_2_, and BDT(DPP-9-BTI)_2_ using benzo[4,5]thieno[2,3-b]indole (BTI) as the end-group donor with a different substitution position. Suspending 8-sbstituted BTI showed a better photovoltaic response than those corresponding with suspending 9-sbstituted BTI due to more and balanced electron and hole mobilities in the 8-substituted position than the 9-substituted position. The PCEs of 4.80% and 3.52% were obtained in the BDT(DPP-8-BTI)_2_ and BDT(DPP-9-BTI)_2_, respectively, with the PC_71_BM acceptor [107]. A new BDT-based molecule, SeBDT-DPP, used diketopyrrolopyrrole (DPP) as the end-group acceptor and a selenophene-based lateral side group incorporated into the core BDT. The optimized device using 1-chloronaphthalene (1-CN) as a solvent additive revealed a PCE of 5.04% as compared to without 1-CN (1.20%). The increment in efficiency after using 1-CN was due to good phase separation and low surface roughness compared to with 1-CN [108]. Heeney et al. reported a new star-shaped molecule, BDT(DPP)_4_, with four conjugated diketopyrrolopyrrole (DPP) arms on the central BDT core. The BDT(DPP)_4_ donor utilized in OPV and a reasonable PCE of 3.9% and 2.5% were achieved when blended with C8-ITIC and PC_71_BM, respectively [109]. Two new BDT-based molecules, M1 and M2, were designed and reported using difluoro-quinoxaline as an electron acceptor and thiophene as a π-bridge. The device based on M1 and M2 showed a poor efficiency of 0.54% and 0.41%, respectively due to low fill factor and low photo response [110]. Tran et al. reported two new BDT molecules using 3-ethyl rhodamine as the end-group acceptor, namely c-SM1 and c-SM2, with one and two central BDT cores, respectively. The extended conjugation length in c-SM2 increased the absorption and Jsc, which resulted in a better efficiency of 0.82% for c-SM2 compared to 0.41% for c-SM1 [111].

Porphyrin and their derivatives have fascinated OPVs as either donors or acceptors owing to their similar properties to chlorophylls, as natural light harvesters in photosynthesis. In 2020, two new molecules, C8T-BDTDP and C8ST-BDTDP using BDT-linked dimeric porphyrin, were reported. The optimized devices C8T-BDTDP:6TIC and C8ST-BDTDP:6TIC exhibited a PCE of 8.73% and 10.39%, respectively. The better PCE of C8ST-BDTDP:6TIC is due to smooth surface morphology and ordered crystalline packing, which resulted in efficient charge dissociation and transportation [112]. Similarly, the same group reported two A-D-A-based porphyrin dimers, C8TEBDT-2P and C8TBDT-2P, with varying π-bridge and in latter porphyrin directly attached with BDT and showed less planar structure than the former. The solar cell devices with IDIC acceptor showed a PCE of 7.46% for C8TEBDT-2P and 2.68% for C8TBDT-2P. The higher PCE in C8TEBDT-2P based devices is mainly due to more light absorption, ordered surface morphology, and more charge carrier mobility than the C8TBDT-2P-based devices. Moreover, the C8TEBDT-2P: IDIC revealed an efficient PCE of 12.3% under indoor (300 lux light-emitting diode) illumination [113]. Two BDT-based new donor molecules, BDT-Qx and BDT-T-Qx, using quinoxaline derivative (Qx) as an acceptor end-group, were designed and reported. The device with BDT-T-Qx showed a higher PCE of 0.59% compared to BDT-Qx (0.52%) owing to extended conjugation with the insertion of thiophene π-bridge [114]. A new D-A-D-A-D-type molecule, 5BDTBDD using benzo [1,2-c:4,5-c’] dithiophene-4,8-dione (BDD) as an electron acceptor unit was designed and compared with a D-A-D-type molecule, 3BDTBDD (with a BDD central core acceptor and BDT donor end). The 5BDTBDD: ITIC cells showed broader absorption, improved mobility, morphology, and crystallinity, which resulted in a better PCE of 7.89% compared to 4.33% for 3BDTBDD: ITIC [115]. More recently, two A-π-D-π-A-type molecules, 3BDT-4 and 3BDT-5, with a BDT trimer (3BDT) as the central core electron donor unit and different electron-withdrawing end groups of 3-hexylrhodanine (3BDT-4) and 2-ethylhexyl cyanoacetate (3BDT-5), were synthesized and reported with a Y6 NFA acceptor. More importantly, the 3BDT-4 and 3BDT-5-based OSCs revealed the efficient PCEs of 5.8% and 10.4%, respectively, without any extra treatment, and with post-treatment no any obvious change occurred, which indicated the effectiveness of molecular design strategies and control of the active layer morphologies [116]. Yuan et al. synthesized and reported a D-A-D-A-D-type donor molecule consisting of alternating BDT units with a 5,6-difl uorobenzo[c][1,2,5] thiadiazole (dFBT) acceptor unit, namely O-BDTdFBT, and applied in OSCs using PC_71_BM acceptor. The photovoltaic results showed that after the addition of high-boiling-point solvent, 1,8-diiodohexane (DIH, 0.5%), as the solvent additive, a remarkable improvement in efficiency from 5.82% to 8.1% was achieved owing to the proficient solving of the inadequate phase-separation (excellent intrinsic mixing of BDTdFBT: PC_71_BM) issue by the generation of homogeneous grains and effective phase separation [117]. Two new BDT-based donors, BDT-O-DPP and BDT-PO-DPP, were reported by using alkoxy and alkoxyphenyl lateral substitution on the central core BDT, respectively. Due to an increase in the torsion angle of the side chain and electronic delocalization in alkoxyphenyl substituted BDT-PO-DPP, the hole mobility was improved and resulted in a PCE of 5.63% compared to the alkoxy substituted BDT-O-DPP (PCE 4.28%) with the PC_61_BM acceptor [118]. Two low-bandgap BDT-based molecules, BDT(DPP-TTHex)_2_ and BDT(DPP-TT)_2_, were designed and reported using different end bithiophene side chains. The X-ray diffraction (XRD) pattern showed that the molecule BDT(DPP-TTHex)_2_ with hexyl at the terminal 5 position of thiophene has a favorite stacking direction along the backbone, while BDT(DPP-TT)_2_ without an alkyl chain at the 5 position showed stacking directions both along and across the backbone, and thus higher crystallinity than the former and an efficiency of 5.12% compared to 2.36% for BDT(DPP-TTHex)_2_ [119]. Zhu et al. reported four novel SMs, BDT(DPP)_2_, BDTT(DPP)_2_, BDT(TPD-DPP)_2_, and BDTT(TPD-DPP)_2_, using diketopyrrolopyrrole (DPP) as an acceptor in the former two molecules and additional thieno [3,4-c]pyrrole-4,6-dione (TPD) as dual acceptor units in the latter two molecules. The OSCs based on BDTT(TPD-DPP)_2_: PC_61_BM showed the best PCE of 4.25% among all, while the PCE of its counterpart BDTT(DPP)_2_ was very poor (0.77%). The transmission electron microscopy (TEM) images showed that the BDTT(TPD-DPP)_2_ had better miscibility and smaller phase separation in the acceptor blend and that their blend film formed a continuous interpenetrating network, which ultimately enhanced the exciton diffusion and charge separation [120]. Yang et al. developed four BDT-based novel molecules, TBCA-CX (X = 2, 4, 6, and 8), with the same conjugated structure, but with variation in their terminal alkyl chains, and fabricated the OSCs with IT-4F acceptor. The molecular orientation of pristine TBCA-CX films changed from edge-on to face-on orientation with the increasing terminal alkyl chains. The more compact molecular packing and favorable face-to-face orientation at the donor/acceptor interfaces observed in the TBCA-C4: IT-4F blend increased the exciton dissociation efficiency and resulted in the best PCE of 9.21%, while its analogs exhibited less than 8% efficiency [121]. Xu et al. reported two novel star-shaped BDT-based molecules, BDT-3Th and BDT-4Th, using alkyl cyanoacetate end-group acceptor. The OSC devices were fabricated with a Y6 acceptor and obtained the PCEs of 3.78% and 5.83% for BDT-3Th and BDT-4Th, respectively. The better PCE in the latter was due to the higher molecular extinction coefficient, deeper HOMO energy level, and much better surface morphology than in BDT-3Th [122].

## 3. Morphology Optimization Techniques

The morphology of active layer material is critical, and it has long been accepted in the literature that the film morphology plays a critical role in organic solar cell performance. After designing the suitable small molecular donors, the next most important aspect of an OSC is the packing of molecules within neat donor and acceptor phases, and their domain sizes should be confined to nearly 30 nm to ensure sufficient exciton diffusion to the donor/acceptor interface for the subsequent exciton dissociation [12,24,26,39]. In order to achieve a high-performing solar cell, much attention has been dedicated to favorable morphology. Below, we discuss morphology optimization using subtle structural modifications and post-treatment techniques.

### 3.1. By Subtle Structure Modification

**Figure 8:**
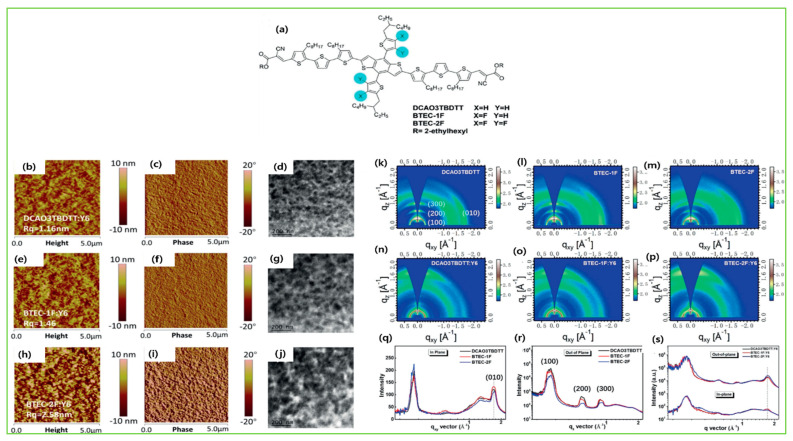
(**a**) Chemical structures of DCAO3TBDTT, BTEC-1F, and BTEC-2F. (**b**,**e**,**h**) AFM height images and (**c**,**f**,**i**) AFM phase images of the blend films for DCAO3TBDTT:Y6, BTEC-1F:Y6, and BTEC-2F:Y6, respectively. (**d**,**g**,**j**) TEM images. (**k**–**m**) 2D GIWAXS images of the neat films after thermal annealing. (**n**–**p**) Two-dimensional GIWAXS images of the blend films processed under optimal conditions. (**q**–**s**) One-dimensional plots of the neat and blend films extracted from 2D GIWAXS images along with out-of-plane and in-plane directions. Adapted with permission from Reference [24]. Copyright 2019, John Wiley and Sons.

**Figure 9:**
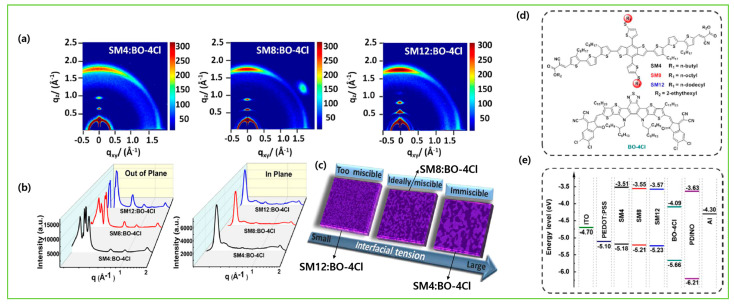
(**a**) Two-dimensional diffraction patterns of the blend films of SM4:BO−4Cl, SM8:BO−4Cl, and SM12:BO−4Cl. (**b**) One-dimensional diffraction profiles in the in-plane and out-of-plane directions for blend films. (**c**) Pictorial illustration of the influence of interfacial tensions on the miscibility. (**d**) Chemical structures of SM4, SM8, SM12, and BO−4Cl. (**e**) Energy levels of all materials used in [26]. Adapted with permission from [26]. Copyright 2021, American Chemical Society.

**Figure 10:**
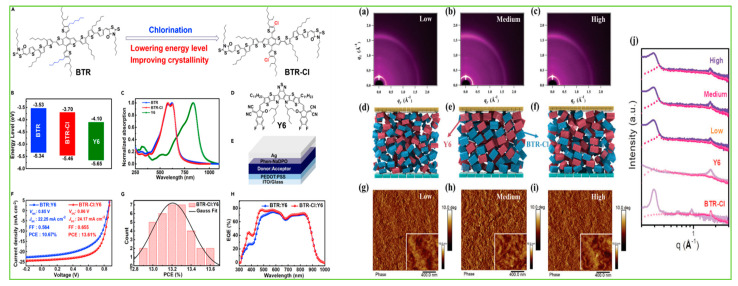
(**A**) Property improvement of BTR by chlorination. (**B**) Energy level diagram of BTR, BTR-Cl, and Y6. (**C**) Film absorption of donor and acceptor materials. (**D**) Molecular structure of Y6. (**E**) Device structure of the ASM OSC. (**F**) J-V curves of the best-performing BTR: Y6 and BTR-Cl: Y6-based ASM OSC. (**G**) Histogram of PCE counts for 35 individual devices with the BTR-Cl:Y6 active layer. (**H**) EQE curves of the best-performing devices. Adapted with permission from [38]. Copyright 2019, CellPress. Two-dimensional GIWAXS patterns of films based on BTR-Cl: Y6 blend of (**a**) low concentration, (**b**) medium concentration, and (**c**) high concentration. The schematic morphology based on BTR-Cl: Y6 blend films of (**d**) low concentration, (**e**) medium concentration, and (**f**) high concentration. AFM phase images with height images inset of BTR-Cl: Y6 blend film of (**g**) low concentration, (**h**) medium concentration, and (**i**) high concentration. (**j**) Corresponding GIWAXS intensity profiles along the in-plane (purple lines) and out-of-plane (red lines) directions. Adapted with permission from [39]. Copyright 2020, John Wiley and Sons.

Here, we attempt to convey more specific information about the subtle modification in chemical structures and their impacts on morphology. The first example is DCAO3TBDTT and its analogous molecules, BTEC-1F and BTEC-2F with Y6 acceptor [24]. The atomic force microscopy (AFM) images showed that the surface roughness increased from DCAO3TBDTT: Y6 (1.16 nm) to BTEC-1F: Y6 (1.46 nm) and BTEC-2F: Y6 (2.58 nm). The same trend was also observed in transmission electron microscopy (TEM) results; the phase images of BTEC-2F: Y6 film exhibited a remarkably coarser and bigger domain size and thus implied a suitable interpenetrating network, exciton dissociation, and charge transport. The grazing-incidence wide-angle X-ray scattering (GIWAXS) result showed that the DCAO3TBDTT and BTEC-1F had the same π-π stacking, while BTEC-2F exhibited more compact π-π stacking distance due to its better planarity and so increased the hole transport behavior, resulting in increases in the fill factor and efficiency (Figure 8b–s). The 2D GIWAXS images for the SM4:BO-4Cl, SM8:BO-4Cl, and SM12:BO-4Cl blend films were utilized to observe the morphology and crystallization. The SM4:BO-4Cl distributed quite a few scattering points, showing that it has high crystallinity and easily forms coarser phase domains, while the SM8:BO-4Cl- and SM12:BO-4Cl-blend films tended to have face-on orientation, indicating the charge transfer in the vertical direction across the active layer. The closest crystal coherence lengths (CCLs) of the π−π stacking and ordered crystal structure was observed based on the SM8:BO-4Cl blend film, benefitted in charge transfer and inhibited molecular recombination, and thus resulted in the best FF and PCE amongst the SM4:BO-4Cl- and SM12:BO-4Cl-blend films (Figure 9a–c) [26]. Replacing the hexyl side chains in the thienyl lateral side group of BTR with chlorine atoms, the BTR-Cl retained the liquid crystalline property with high crystallinity and the device with Y6 acceptor formed a more textured surface, with wrinkle-like microstructures, and a well-balanced phase separation and optimal film morphology were obtained and yielded a higher efficiency of 13.67% compared with the control device, BTR (10.67%) (Figure 10A–H) [38]. With further device optimization, Tang et al. demonstrated that concentration-induced delicate fine-tuning of the morphological strategy of BTR-Cl:Y6 exhibited an efficient PCE of 14.7% (Figure 10a–i) [39].

**Figure 11:**
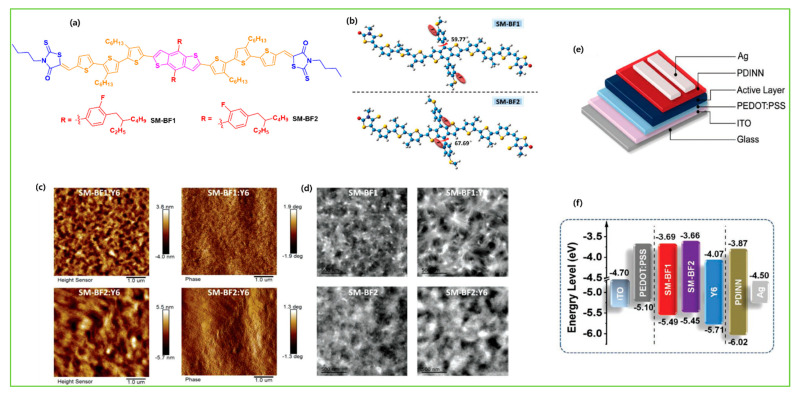
(**a**) Chemical structures of the SMDs SM−BF1 and SM−BF2. (**b**) Optimized molecular structures of the SMDs obtained from the DFT-based theoretical calculations. (**c**) AFM height images and phase images of the SM-BF1:Y6 and SM−BF2:Y6-blend films. (**d**) TEM images of the SM−BF1 and SM−BF2 neat films and the SM−BF1:Y6 and SM−BF2:Y6 blend films. (**e**) Device structure and (**f**) energy level diagram of the related materials used in the SM-OSCs. Adapted with permission from [14]. Copyright 2021, John Wiley and Sons.

**Figure 12:**
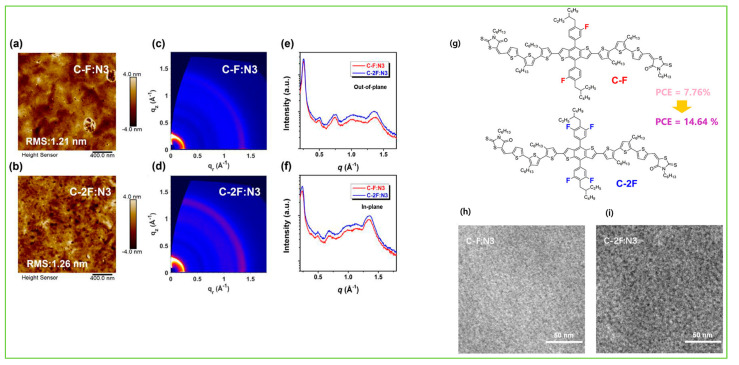
(**a**,**b**) AFM height images of C-F:N3 and C-2F:N3 blend films. (**c**,**d**) GIWAXS two-dimensional diffraction patterns of C-F:N3 and C-2F:N3. (**e**,**f**) In-plane and out-of-plane line-cut profiles of the two-dimensional GIWAXS data of C-F:N3 and C-2F:N3 blend films. (**g**) Chemical structures of C-F and C-2F. (**h**,**i**) TEM images of C-F: N3 and C-2F: N3 blend films, respectively. Adapted with permission from [49]. Copyright 2022, American Chemical Society.

**Figure 13:**
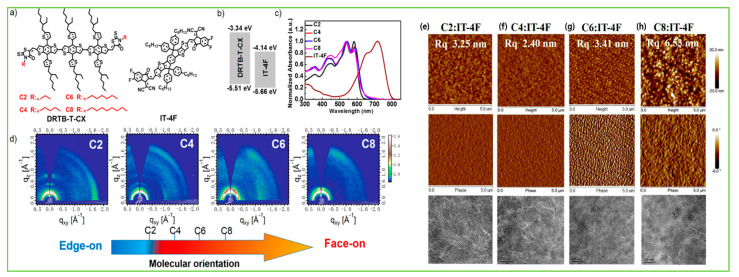
(**a**) Molecular structure of DRTB−T-CX and IT−4F. (**b**) Energy level diagram of the donor and acceptor materials. (**c**) Normalized thin-film UV−vis absorption spectra of DRTB−T-CX and IT−4F. (**d**) Two-dimensional GIWAXS patterns of pristine C2, C4, C6, and C8. (**e**–**h**) AFM height and phase images and brightfield (BF)TEM images of films spin coated from the DRTBTCX:IT4F blends with SVA treatments. Adapted with permission from [104]. Copyright 2018, American Chemical Society.

**Figure 14:**
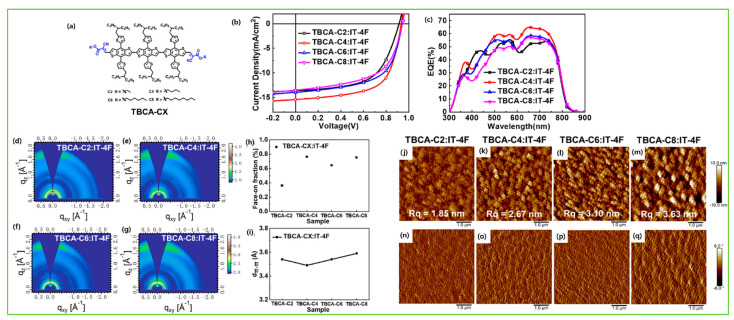
(**a**) Chemical structure of TBCA-CX. (**b**) J-V curves. (**c**) EQE spectra of TBCA-CX: IT−4F BHJ devices. (**d**–**g**) Two-dimensional GIWAXS patterns of TBCA-CX: IT−4F blend films. (**h**) Face-on orientation ratios for the TBCA-CX: IT−4F blend films. (**i**) Out-of-plane (010) dπ–π for the TBCA-CX: IT−4F blend films. (**j**–**q**) AFM height and phase images of the TBCA-CX:IT−4F blend films. Adapted with permission from [121]. Copyright 2022, Elsevier.

**Figure 15:**
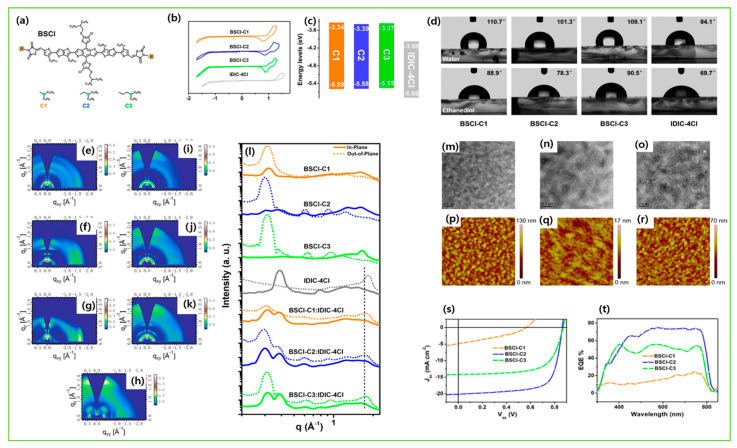
(**a**) Chemical structure of small-molecular donors. (**b**) Cyclic voltammograms (CV). (**c**) Energy level of the related donor and acceptor materials used in the SM-OSCs. (**d**) Contact angles of water and ethanediol droplets on BSCl-C1, BSCl-C2, and BSCl-C3 and IDIC-4Cl pristine films. (**e**–**h**) Two-dimensional GIWAXS patterns of donors BSCl-C1, BSCl-C2, and BSCl-C3 and acceptor IDIC-4Cl pure films. (**i**–**k**) Two-dimensional GIWAXS patterns of blended films under optimized conditions and (**l**) one-dimensional GIWAXS curves of BSCl-C1, BSCl-C2, BSCl-C3, and IDIC-4Cl pure and blended films under optimized conditions. (**m**–**o**) TEM images of blended films based on different donors BSCl-C1, BSCl-C2, and BSCl-C3 and (**p**–**r**) corresponding AFM height images. (**s**) Optimized J-V curves for the three systems and (**t**) EQE spectra of the corresponding devices. Adapted with permission from [123]. Copyright 2020, American Chemical Society.

The introduction of fluorine atoms on the conjugated side chains of the BDT central unit improve interchain interactions and crystalline properties, which is beneficial for achieving high hole mobility and PCE. By alternating the fluorine atom position on the conjugated BDT side chains, the steric effect, twisted conformation in molecular backbone, and molecular aggregation were observed in SM-BF1 and SM-BF2. The dihedral angle between the phenyl-conjugated side chain and the main backbone of ortho-fluorinated SM-BF1 is 59.77°, smaller than that of the meta position of SM-BF2, 67.69° (Figure 11a,b). The TEM images (Figure 11d) showed that SM-BF2 film has a little larger, relatively dispersed cluster structure, while the SM-BF1 film has a smaller particle size and tighter packing. The SM-BF1 molecule possessed less twisted molecular configuration and formation of tight molecular packing. The SM-BF1:Y6-blend film exhibited more uniform fiber aggregation and tended to form appropriate nano-phase interpenetrating networks, suitable for better exciton extraction and carrier transport, thus effectively transferring the charge and leading to a superior PCE of 15.71% compared to the SM-BF2:Y6 blend (PCE 10.23%) [14]. Cai et al. recently demonstrated that symmetrically difluorinated phenyl group attached to the BDT core in the C-2F molecule has the potential to increase the π-π stacking distance and alter the molecular packing behavior and crystallinity compared with the asymmetrically monofluorinated phenyl group attached to the BDT core in C-F molecule. The AFM images showed that both molecules had nearly the same RMS values as the N3 acceptor blends (Figure 12a,b) but the GIWAXS measurement (Figure 12c–f) for C-2F:N3 film indicated its larger crystal grain size and CCL compared to C-F:N3 film, facilitating the charge transport three-dimensionally and thus increasing the PCE from 7.76% to 14.64% for C-F to C-2F [49]. The longer alkyl chain is employed to improve solubility and film quality. Moreover, the alkylation on the end group significantly affected the molecular aggregation and changed the molecular orientation. Yang et al. showed that the variation of the alkyl chains (from C2 to C8) on the rhodamine end-group have nearly the same photoelectric properties but varied molecule orientation. The molecular orientation slowly shifted from edge-on dominance to face-on domination (DRTB-T-C2 to BRTB-T-C8). Between them, the butyl (C-4)-containing chains in the rhodamine unit revealed face-on orientation and the best crystallinity in their blend films. Conversely, the decrease in the face-on fraction of C6 compared with those of C4 and C8 is because of the creation of the preferred orientation at 30° with respect to the surface of the substrate. In the solid thin-film state, the face-on orientation was accomplished by increasing the crystal coherence length (CCL) of π-π stacking and resulted in enhancing the carriers’ mobility. It is well-known that Jsc is strongly dependent on the domain size. The DRTB-T-C4:IT-4F system had the smallest domain size (Figure 13e,h), which led to a larger interfacial area and thus an efficient exciton dissociation efficiency, further leading to an excellent PCE of 11.24%, whereas the C8 system has the largest domain size, which might be too large for efficient exciton separation, and so the Jsc value and performance decreased in the DRTB-T-C8:IT-4F system [104]. Similarly, in 2022, the same group replaced the rhodamine acceptor with alkylcyanoacetate (alkyl = C2 to C8) in DRTB-T-CX molecules and obtained TBCA-CX. All the blends of TBCA-CX with IT-4F acceptor showed strong diffractions in the out-of-plane direction, suggesting dominant face-on-orientation (Figure 14d–i). The face-on-orientation increased with the extension of alkyl chain length. The TBCA-C4: IT-4F blend film exhibited the smallest π-π stacking distance of 3.49 Å, resulting in stronger intermolecular aggregation between donor and acceptor, while the π-π stacking distance of the TBCA-C6 and TBCA-C8 blends with IT-4F acceptor increased from 3.54 Å to 3.59 Å, respectively. AFM results also indicated that the RMS values gradually increased with the extension of the length of alkyl chain (Figure 14j–q). Suitable domain size and proper aggregations of TBCA-C4 with IT-4F acceptor provided an efficient channel for charge transport. More compact molecular packing, higher face-on-orientation ratio of TBCA-C4:IT-4F, and suitable aggregation of donor and acceptor resulted in a better PCE of 9.21% [121]. The alkyl chain length and position are an effective approach for molecular packing and orientation. Moreover, controlling the alkyl chains in the end-group of the donor molecule is highly expected to alter the nanostructures of the active layer by modifying with acceptor molecules. Wu et al. showed three molecules, BSCl-C1/C2/C3 with branching points (R-C1, R-C2, and R-C3) in alkyl terminal chains, offering some hints about their miscibility with acceptor IDIC-4Cl. The 2D-GIWAXS patterns of donor pristine showed edge-on molecular packing and after mixing, the position and intensity of the diffraction peaks changed, but the original packing direction was maintained (Figure 15e–k). Due to high ordering, strong molecular crystallinity, and intermolecular interaction with acceptor, a sufficient phase separation and proper domain size were observed in the BSCl-C2-blended film and led to a higher PCE of 12.4% compared to the BSCl-C1- and BSCl-C3-blended films [123].

### 3.2. By Post Treatment (TA, SVA, and Solvent Additives)

Post-treatment techniques, which lead to a delicate balance between miscibility and crystallinity of active layers, are widely used in OSCs to improve the device performance. The top-performing BHJ-OSC devices revealed efficient PCEs by optimizing the morphology through post-treatments either through thermal annealing (TA), solvent vapor annealing (SVA), and solvent additive alone, or by the combination of the TA, SVA, and solvent additives [25,32,36,46,78,83,124,125].

**Figure 16:**
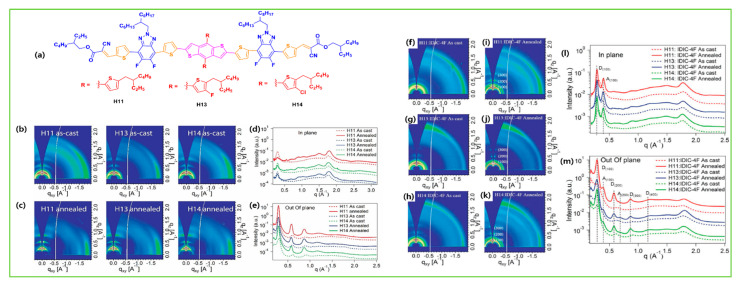
(**a**) Chemical structure of H11, H13, and H14 donors. (**b**,**c**) Two-dimensional GIWAXS patterns without and with thermal annealing. (**d**,**e**) In-plane and out-of-plane 1D line cuts of the neat donor films without and with thermal annealing. (**f**–**k**) Two-dimensional GIWAXS patterns. (**l**,**m**) In-plane and out-of-plane scattering profiles of the blend films without and with thermal annealing. Adapted with permission from [83]. Copyright 2020, John Wiley and Sons.

**Figure 17:**
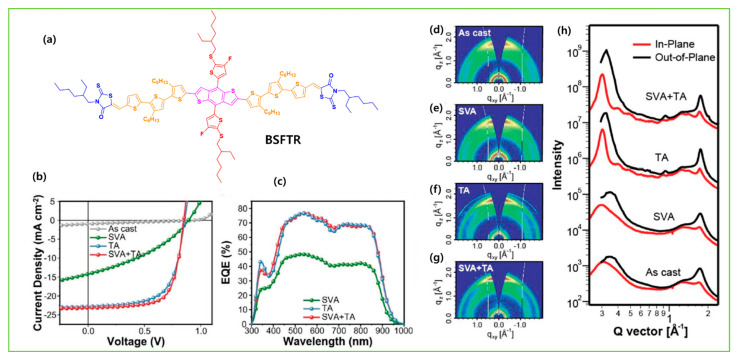
(**a**) Chemical structure of BSFTR donor. (**b**,**c**) J-V and EQE curves for SMSCs based on BSFTR and Y6 with different post-treatments under AM 1.5 G irradiation. (**d**–**g**) Two-dimensional GIWAXS patterns and (**h**) out-of-plane and in-plane scattering profiles of the blend films without and with post-treatments. Adapted with permission from [46]. Copyright 2019, John Wiley and Sons.

**Figure 18:**
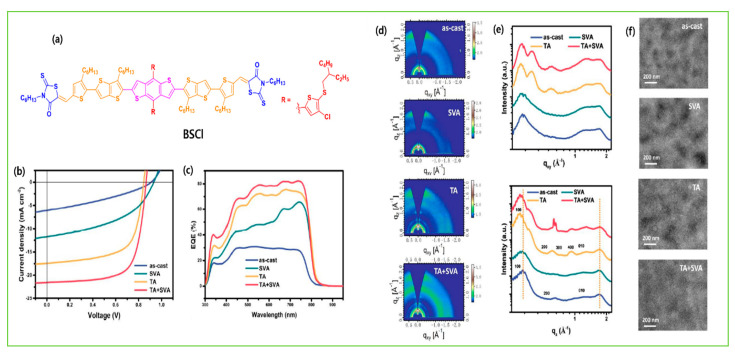
(**a**) Chemical structure of BSCl donor. (**b**,**c**) J-V and EQE curves for SMSCs based on BSCl:IDIC-4Cl blend films with different post-treatments under AM 1.5 G irradiation. (**d**) Two-dimensional GIWAXS graphs with different post-treatment. (**e**) Corresponding curves of 2D GIWAXS patterns and (**f**) TEM images. Adapted with permission from [124]. Copyright 2020, Royal Society of Chemistry.

TA typically heats the BHJ active layer on a hot plate for several minutes between temperature from 70 °C to 200 °C as the thermal annealing generally leads to enhance the crystallinity, and phase separation through the increased molecular kinetic motion. Bin et al. showed the 2D GIWAXS patterns differences between molecular packing and crystallinity of the as-cast and thermally annealed molecules H11, H13, and H14 with IDIC-4F acceptor. The integrated peak intensity increased after annealing, and more intense diffraction peaks due to lamellar stacking in OOP and π–π stacking in IP directions were observed, confirming that the thermal annealing induced molecular aggregation and enriched the edge-on orientation of the neat donors. After blending, the neat film and thermally annealed film showed a distinct enhanced diffraction intensity in the OOP direction of the H11- and H14-based blends (Figure 16a–m). Due to the presence of Cl atoms on the side chain of H14, strong intermolecular interaction and a high degree of aggregation were observed, which further reached appropriate phase separation and high relative domain purity and led to an efficient PCE of 12.1% [83]. In many high-molar-mass systems, thermal treatments alone are not very effective and very long anneal times are often required. To combat such long annealing periods, SVA has been shown to be generally much more effective. SVA is a widely used technique for controlling the morphology of active layer materials in BHJ-OSCs. In SVA, the as-cast active layer is exposed to solvent vapor in a relatively sealed environment for several seconds, forming a swollen and mobile layer to direct the self-assembly and subsequent solvent evaporation, and an active blend film forming more well-organized nanostructures and gaining high mobility. Yue et al. optimized the BSFTR:Y6 BHJ active layer by combining the TA and SVA techniques. The GIWAXS result showed that after SVA, the diffraction peaks became slightly sharper and promoted the molecular crystallinity of BSFTR:Y6, while TA dramatically enhanced the crystallinity with an extra peak and the ordering of Y6, and thus electron mobility increased. When the SVA and TA treatments are combined, there is a small decrease in the crystallinity as compared with the TA-treated blend, which indicates that it is difficult to reach the same high ordering degree as the solely TA-treated film. More importantly, the SVA + TA combination can make mobilities more balanced, which reduced the charge recombination, and thus the BSFTR: Y6 system led to an efficient PCE of 13.69% (Figure 17a–h) [46]. Zhang et al. designed a novel molecule BSCl, and after combining TA and SVA, an efficient PCE of 13.03% was achieved with IDIC-4Cl acceptor. The GIWAXS images revealed that TA+SVA diffraction peaks have a high-order and increased calculated crystal coherence lengths (CCL) of donor crystallite in the out-of-plane direction of blend films. The TEM results also confirmed that the blend films exhibited a more bicontinuous interpenetrating network with phase separation of nearly 20 nm, and which suitably increase exciton efficiency (Figure 18a–f) [124].

## 4. Conclusions and Outlook

ASM-OSCs have drawn increasing attention due to their easy synthesis, purification, and batch-to-batch reproducibility, and a maximum PCE of 17% has been achieved. In future, the main challenges that need to be resolved are as follows.

The most essential requirement for the realization of high efficiency is the active layer material. It is suggested that the π-electron of the BDT unit could delocalize to the 2D side-chains resulting in the enlarged π-conjugation, and thus better intermolecular interactions and enhanced charge transport ability in the films. Introducing functional substitution, such as sulfur, silicon, and/or halogen atoms (F and Cl), is an effective method to tune the properties of the photovoltaic materials. Due to their large electronegativity, halogen atoms could be used to tune the HOMO/LUMO levels and crystalline properties of photovoltaic materials. Moreover, the combined chlorination and sulfuration strategies may effectively deepen the HOMO level and enhance high crystallinity, which increases Voc and carrier mobility. A symmetrical phenyl side chain unit has almost the same energy levels and a similar absorption spectrum to a thienyl side chain unit, but adopting phenyl side chain units may effectively improve the crystalline property and increase π-π stacking. The end alkyl chain has a negligible effect on their molecular orbital levels and optical absorptions, but gradually changes from the edge-on dominance to the face-on dominance and thus leads to higher charge transportation. In the development of new molecules, chemists need balance between solubility, molecular miscibility with donor and/or acceptor, phase aggregation tendency in film state, potential carrier mobilities, and special attention on energetics and optical properties. Currently, each class of material fulfils some of above merits but also limitation on others. Active layer morphology is a critical factor affecting the performance such as charge transport and collection, energy loss, and device stability of SM-OSCs. Active layer morphology is very sensitive to processing conditions. The widely used techniques to fine-tune the morphology are TA, SVA, and solvent additives. The optimized morphologies should possess characteristics such as interpenetrated blend networks with suitable phase separation and domain size, and face-on molecular packing. Moreover, morphologies are very sensitive to the time of SVA, temperature and time of TA, which raises problems for future commercial applications. The best answer to solve this issue is developing high-performance as-cast devices, which mainly require the further involution of active layer materials.

The theoretical Shockley–Queisser (SQ) value for energy loss (E_loss_) in OSC is (0.25–0.3 eV), while the state-of-the art OSC still suffer from a large E_loss_ (0.5 to 0.6 eV), which further limits the PCE of OSCs. The non-radiative recombination energy loss (ΔE3) is the main barrier to further reduce the E_loss_, so more attention is needed on how to reduce (ΔE3) for higher Voc without sacrificing FF and JSC. Molecules with smaller HOMO/LUMO differences can lead to stronger hybridization between the localized excitation state and the charge transfer (CT) state. In OSC cells, enhancing the luminescence of the CT state decreases nonradiative recombination and improves Voc. Photoluminescence quantum yield (PLQY) of the active material can determine the lower limit of ΔE3 and an increase of one order of magnitude of EQE_EL_, and (ΔE3) will decrease by ~60 meV. To understand the better relationship between molecular structure, intermolecular interactions, and the EQE_EL_ of a device, sensitive methods must be developed to detect, characterize, and establish these relationships. The understanding of such a relationship could help to rationally design materials to achieve ultra-low ΔE3 with effective exciton dissociation and charge transport in OPV cells. The compact molecular packing with a 3D packing network and strong electronic coupling between adjacent molecules would effectively decrease the ΔE3 and thus a greater PCE can be realized in OSC. The conjugated skeleton of organic materials is strongly correlated with the energy loss of the devices in OSCs. In short, the two main factors involved in large E_loss_ are, firstly, the existence of considerable energy offset between the D/A materials along with the CT state, and secondly the relatively large non-radiative recombination energy loss. Identifying and optimizing these loss pathways would further alleviate the efficiency of OSCs and be comparable to perovskite and inorganic devices.

In achieving high-stability OSCs, thermal- and photostability are always the biggest challenges. In the conventional fullerene systems, the dimerization of the fullerene, as well as the diffusion of the fullerene component into the SMDs, are the main cause of device instability. Recent results suggest the better stability of NFA-based devices compared with fullerene-based control devices, which further strengthens the future use of OSCs. The thermal stability of SMDs and NFAs is excellent, with a typical decomposition degree over 300 °C, which is important for the long-term stability. Though a high efficiency over 15% was achieved for the NF-SMOSCs, less work has focused on their stability. Understanding the nature of degradation mechanisms in OSCs is crucial to finding the solutions to improve their stability. We recommend that more attention is paid to this issue in the future to find a way to conquer this obstacle.

For industrial scale production of OSCs, the active layer thickness should be enough for large-scale printing without breaking the film. Until now, most nonfullerene BHJ-OSCs contain ~100 nm active layer thickness because of the relatively low electron mobility as compared to fullerene. Developing new NFAs with high mobility can mitigate this issue. Instead of using current halogenated solvents, which are environmentally unfriendly and toxic to human health, developing eco-friendlier nonhalogenated solvents to process highly efficient and air-stable devices is a major challenge in OSCs. The simple and low-cost synthetic route is very important to competing with other solar energy technologies.

It will speed up the industrialization of OSCs if these aforementioned challenges are overcome. We believe that this review will encourage further research on the rational design and synthesis of novel BDT-based organic small molecules with cost-effective, better performance, and high-stability organic solar cells.

## Figures and Tables

**Table 1:** Optoelectronic properties and device parameters for BDT-based ASM OSCs via lateral side chain engineering.

Donor	Acceptor	E_g_ (eV)	HOMO/LUMO (eV)	*µ*_h_ (cm^2^V^−1^s^−1^)	V_OC_ (V)	J_SC_ (mAcm^−2^)	FF (%)	PCE (%)	Ref.
DCAO3T(BDT)3T	PC_61_BM	1.83	−5.11/−3.54	1.51 × 10^−4^	0.93	9.77	59.9	5.44	[19]
TB-BDT6T	PC_61_BM	1.76	−5.31/−3.63	5.38 × 10^−5^	0.85	6.87	58.7	3.59	[20]
ST-BDT6T	PC_61_BM	1.75	−5.40/−3.67	8.19 × 10^−5^	0.96	8.47	60.5	4.98	[20]
TT-BDT6T	PC_61_BM	1.78	−5.35/−3.64	1.94 × 10^−4^	0.97	9.40	61.8	5.79	[20]
DCAO3TBDT	PC_61_BM	1.84	−5.04/−3.24	1.38 × 10^−4^	0.95	8.00	60.0	4.56	[21]
DR3TBDT	PC_71_BM	1.74	−5.02/−3.27	2.47 × 10^−4^	0.93	12.21	65.0	7.38	[21]
DCA3TBDTP	PC_61_BM	1.82	−5.25/−3.43	2.74 × 10^−4^	0.90	7.88	63.6	4.51	[22]
SMD1	PC_71_BM	1.82	−5.23/−3.47	1.40 × 10^−4^	0.96	6.95	53.8	3.59	[23]
SMD2	PC_71_BM	1.83	−5.19/−3.45	1.04 × 10^−4^	0.95	5.11	64.4	3.13	[23]
SMD3	PC_71_BM	1.74	−5.40/−3.72	1.95 × 10^−4^	0.94	8.00	62.2	4.67	[23]
DR3TBDTT	Y6	1.92^cv^	−5.25/−3.33	4.60 × 10^−4^	0.80	21.71	60.9	10.64	[24]
BTEC-1F	Y6	2.00^cv^	−5.37/−3.37	4.17 × 10^−4^	0.87	21.21	61.3	11.33	[24]
BTEC-2F	Y6	2.01^cv^	−5.39/−3.38	5.43 × 10^−4^	0.85	21.55	72.3	13.34	[24]
BT-2F	Y6	2.00^cv^	−5.40/−3.40	3.93 × 10^−4^	0.85	22.38	72.27	13.80	[25]
BT-2F	N3	-	-	-	0.84	23.81	70.22	14.09	[25]
SM4	BO-4Cl	1.83	−5.18/−3.51	3.51 × 10^−4^	0.76	18.18	46.8	6.44	[26]
SM8	BO-4Cl	1.82	−5.21/−3.55	3.93 × 10^−4^	0.85	21.23	72.5	13.11	[26]
SM12	BO-4Cl	1.82	−5.23/−3.57	2.25 × 10^−4^	0.84	19.20	65.9	10.59	[26]
BT-R_O_-Cl	Y6	1.84	−5.41/−3.57	2.46 × 10^−4^	0.86	22.50	68.6	13.35	[27]
BT-R_EH_-Cl	Y6	1.83	−5.41/−3.58	2.81 × 10^−4^	0.86	22.93	69.8	13.90	[27]
H21	IDIC	1.81	−5.38/−3.63	2.49 × 10^−4^	0.90	13.00	65.6	7.62	[28]
H22	IDIC	1.89	−5.39/−3.59	4.26 × 10^−4^	0.94	15.38	71.2	10.29	[28]
BDTF-CA	IDIC	1.90	−5.44/−3.53	5.4 × 10^−4^	0.99	12.09	51.14	6.12	[29]
BDTF-CA	IDIC-2F	-	-	1.9 × 10^−4^	0.94	12.69	58.07	9.11	[29]
BDTF-CA	IDIC-4F	-	-	6.3 × 10^−4^	0.88	14.96	63.95	8.42	[29]
SM	IDIC	1.92	−5.25/−3.33	3.39 × 10^−4^	0.91	15.18	67.8	9.39	[30]
SM-Cl	IDIC	2.08	−5.42/−3.34	2.58 × 10^−4^	0.97	12.61	63.08	7.73	[30]
SM:SM-Cl (1.8:0.2)	IDIC	-	-	4.01 × 10^−4^	0.92	16.05	69.58	10.29	[30]
DR3TBDTT	PC_71_BM	1.72	−5.02/−3.27	2.88 × 10^−4^	0.93	13.17	66.2	8.12	[31]
DR3TBDTT-HD	PC_71_BM	1.76	−5.06/−3.29	1.52 × 10^−4^	0.96	11.92	59.4	6.79	[31]
DR3TBDT2T	PC_71_BM	1.76	−5.07/−3.29	3.29 × 10^−4^	0.92	12.09	72.1	8.02	[31]
DR3TSBDT	PC_71_BM	1.74	−5.07/−3.30	6.13 × 10^−4^	0.91	14.45	73.0	9.95	[32]
BDTT-S-TR	PC_70_BM	1.73	−5.18/−3.25	6.57 × 10^−4^	0.97	13.45	70.5	9.20	[33]
BDTT-TR	PC_70_BM	1.74	−5.17/−3.39	5.48 × 10^−4^	0.93	11.75	68.1	7.44	[34]
BDTT-O-TR	PC_70_BM	1.74	−5.14/−3.34	3.70 × 10^−4^	0.90	11.03	65.5	6.50	[34]
DRBDT-TT	PC_71_BM	1.78	−5.13/−3.33	5.41 × 10^−4^	0.92	13.12	72.0	8.70	[35]
DRBDT-STT	PC_71_BM	1.80	−5.15/−3.34	4.74 × 10^−4^	0.91	12.40	71.0	8.01	[35]
DR3TBDTT	PC_71_BM	-	-	6.57 × 10^−4^	0.88	14.21	76.0	9.58	[36]
DR3TDOBDT	PC_71_BM	1.79	−5.08/−3.27	4.08 × 10^−4^	0.94	12.56	70.0	8.26	[37]
BTR	Y6	1.78	−5.34/−3.53	3.01 × 10^−4^	0.85	22.25	56.4	10.67	[38]
BTR-Cl	Y6	1.78	−5.46/−3.70	2.72 × 10^−4^	0.86	24.17	65.5	13.61	[38]
BTR-Cl	Y6	-	-	8.51 × 10^−5^	0.83	23.66	74.7	14.7	[39]
BTR-Cl	PC_71_BM:Y6	-	-	9.78 × 10^−4^	0.84	23.75	77.1	15.34	[13]
DRBDT-TVT	PC_71_BM	1.75	−5.11/−3.41	3.6 × 10^−4^	0.88	10.73	72.76	6.87	[40]
DRBDT-STVT	PC_71_BM	1.76	−5.14/−3.43	3.4 × 10^−4^	0.91	10.25	73.61	6.84	[40]
DRBDT-TVT	IDIC	-	-	1.4 × 10^−4^	0.84	12.22	64.58	6.63	[40]
DRBDT-STVT	IDIC	-	-	5.0 × 10^−5^	0.89	10.93	67.17	6.51	[40]
BDTTS-F-R	PC_71_BM	1.76	−5.28/−2.82	3.24 × 10^−4^	0.95	14.31	68.9	9.37	[41]
DTTS-Cl-R	PC_71_BM	1.77	−5.35/−2.84	2.79 × 10^−4^	0.96	14.92	75.3	10.78	[41]
BDTTS-Br-R	PC_71_BM	1.78	−5.40/−2.87	1.85 × 10^−4^	0.98	13.85	63.1	8.55	[41]
DRBDTCO	PC_71_BM	1.73	−4.94/−3.30	6.5 × 10^−4^	0.87	12.54	75.0	8.18	[42]
dDRBDTCO	PC_71_BM	1.80	−5.02/−3.30	5.9 × 10^−4^	0.89	10.88	73.0	7.07	[42]
BTR-TIPS	PC_71_BM	1.84	−5.42/−3.13	2.8 × 10^−4^	0.94	8.4	62.0	5.0	[43]
BTR-TE	PC_71_BM	1.78	−5.39/−3.33	9.4 × 10^−4^	0.90	14.0	70.0	9.0	[43]
BTR-H	PC_71_BM	1.76	−5.32/−3.29	3.9 × 10^−4^	0.87	10.1	58.0	5.1	[43]
BTR-EH	PC_71_BM	1.73	−5.32/−3.34	7.2 × 10^−4^	0.89	11.8	67.0	7.0	[43]
BTR	Y6	-	-	3.81 × 10^−4^	0.83	22.1	61.0	11.0	[44]
BTR-TE	Y6	-	-	2.92 × 10^−4^	0.84	23.4	67.0	13.2	[44]
BTR-TIPS	Y6	-	-	5.89 × 10^−5^	0.83	18.5	54.0	8.3	[44]
PM6:BTR(0.9:0.1)	Y6	-	-	-	0.84	24.6	76.0	15.7	[44]
PM6:BTR-TE(0.9:0.1)	Y6	-	-	-	0.84	24.5	78.0	16.1	[44]
PM6:BTR-TIPS(0.9:0.1)	Y6	-	-	-	0.82	24.1	71.0	14.0	[44]
BTR	NITI	-	-	2.91 × 10^−4^	0.95	15.02	48.69	6.82	[45]
BTR	PC_71_BM	-	-	4.43 × 10^−4^	0.90	13.80	72.86	9.03	[45]
BTR	PC_71_BM:NITI	-	-	6.14 × 10^−4^	0.94	19.50	73.83	13.63	[45]
BSFTR	Y6	1.98^cv^	−5.59/−3.61	6.43 × 10^−4^	0.85	23.16	69.66	13.69	[46]
BDT3TR-SF	NBDTP-F_out_	1.81	−5.37/−2.85	1.80 × 10^−4^	0.80	21.40	64.6	11.02	[47]
BDT3TR-SF	NBDTP-F_in_	-	-	3.27 × 10^−5^	0.87	0.07	32.4	0.01	[47]
BTR	BO-4Cl	1.82^cv^	−5.34/−3.52	1.4 × 10^−3^	0.83	18.93	72.0	11.3	[12]
B1	BO-4Cl	1.86^cv^	−5.37/−3.51	2.3 × 10^−3^	0.83	24.41	75.0	15.3	[12]
SM-BF1	Y6	1.75	−5.49/−3.69	1.42 × 10^−4^	0.85	26.64	69.7	15.71	[14]
SM-BF2	Y6	1.77	−5.45/−3.66	4.86 × 10^−5^	0.80	20.21	63.1	10.23	[14]
S35	Y6	1.80	−5.37/−3.57	4.11 × 10^−4^	0.85	22.54	62.35	11.95	[48]
S35−1Si	Y6	1.80	−5.43/−3.63	7.00 × 10^−4^	0.86	22.93	61.84	12.19	[48]
S35−2Si	Y6	1.81	−5.38/−3.57	8.20 × 10^−4^	0.83	23.59	68.80	13.50	[48]
C-F	N3	1.88	−5.29/−3.53	2.81 × 10^−4^	0.79	20.51	47.52	7.76	[49]
C-2F	N3	1.90	−5.36/−3.54	2.70 × 10^−3^	0.85	24.87	69.33	14.64	[49]
PM6:Y6	PC_71_BM	-	-	1.04 × 10^−3^	0.85	26.37	76.0	17.00	[50]
PM6:CNS-6-8	Y6:PC_71_BM	1.93^cv^	−5.55/−3.62	1.21 × 10^−3^	0.87	26.43	78.8	18.07	[50]
SW1	Y6	1.79	−5.42/−3.43	4.11 × 10^−4^	0.81	25.09	63.8	12.90	[51]
SW2	Y6	1.81	−5.47/−3.45	3.28 × 10^−4^	0.84	25.10	74.0	15.51	[51]
BO-1	BTP-eC9	1.72	−5.30/−3.51	1.03 × 10^−3^	0.85	25.56	77.74	16.79	[52]
HD-1	BTP-eC9	1.73	−5.31/−3.46	1.14 × 10^−3^	0.84	26.04	78.46	17.19	[52]
OD-1	BTP-eC9	1.73	−5.36/−3.48	0.99 × 10^−3^	0.83	25.49	71.89	15.18	[52]

**Table 2:** Optoelectronic properties and device parameters for BDT-based ASM OSCs via end group engineering.

Donor	Acceptor	E_g_ (eV)	HOMO/LUMO (eV)	*µ*_h_ (cm^2^V^−1^s^−1^)	V_OC_ (V)	J_SC_ (mAcm^−2^)	FF (%)	PCE (%)	Ref.
SMPV1	PC_71_BM	1.77	−5.51/−3.64	3.3 × 10^−4^	0.94	12.5	69.0	8.1	[53]
D(T_3_-DCRD)-BDT	PC_61_BM	1.62	−5.39/−2.84	5.07 × 10^−5^	0.93	2.44	49.0	1.10	[54]
D(T_3_-DCRD)-BDTT	PC_61_BM	1.61	−5.46/−2.83	6.22 × 10^−4^	0.96	3.69	55.0	1.94	[54]
DRT3-BDT	PC_71_BM	1.74	−5.42/−3.54	8.68 × 10^−5^	0.90	11.92	63.0	6.76	[55]
DTT3-BDT	PC_71_BM	1.84	−5.38/−3.44	2.94 × 10^−5^	0.86	10.52	58.0	5.25	[55]
DOO3OTTBDT	PC_71_BM	1.76	−5.19/−3.46	1.4 × 10^−4^	0.94	8.0	70.0	5.26	[56]
DOP3HTTBDT	PC_71_BM	1.77	−5.11/−3.37	1.1 × 10^−4^	0.87	9.94	65.0	5.64	[56]
BDT3SCNCOO	PC_71_BM	1.84	−5.08/−3.47	1.2 × 10^−4^	0.89	9.98	72.0	6.4	[57]
BDT3SCNCO	PC_71_BM	1.76	−5.18/−3.46	4.0 × 10^−5^	0.92	10.2	65.0	6.4	[57]
BDT3SCNSOO	PC_71_BM	1.85	−5.11/−3.46	1.4 × 10^−6^	0.93	6.1	53.0	3.0	[57]
BTID-0F	PC_71_BM	1.71	−4.91/−3.20	4.70 × 10^−4^	0.93	14.0	64.0	8.30	[58]
BTID-1F	PC_71_BM	1.70	−4.98/−3.28	6.4 × 10^−4^	0.94	15.3	72.0	10.4	[58]
BTID-2F	PC_71_BM	1.68	−5.05/−3.37	1.4 × 10^−3^	0.95	15.7	76.0	11.3	[58]
2F-C4C6	IDIC	1.82 ^cv^	−5.19/−3.37	7.26 × 10^−5^	0.84	12.05	57.2	6.21	[59]
2F-C6C8	IDIC	1.83 ^cv^	−5.24/−3.41	8.72 × 10^−5^	0.90	13.98	65.2	8.23	[59]
V-BDT	PC_71_BM	2.02	−5.34/−3.64	-	0.89	6.88	61.0	3.73	[60]
BTR-OH	PC_71_BM	1.82	−5.49/−3.45	1.4 × 10^−5^	0.90	13.56	65.3	8.0	[61]
BTR:BTR-OH(0.8:0.2)	PC_71_BM	-	-	6.8 × 10^−5^	0.93	14.62	74.2	10.14	[61]
BDT-1	PC_71_BM	1.73	−5.28/−3.25	6.9 × 10^−5^	0.90	11.4	52.9	5.46	[62]
BDT-2	PC_71_BM	1.78	−5.27/−3.22	7.0 × 10^−6^	0.82	8.3	43.9	2.99	[62]
BDT-3	PC_71_BM	1.96	−5.25/−2.90	1.0 × 10^−8^	0.55	2.6	27.0	0.38	[62]
DR3TBDTT	PC_71_BM	1.77	−5.02/−3.27	3.53 × 10^−4^	0.90	13.37	74.8	9.09	[63]
DCAO3TBDTT	IDIC	1.88	−5.24/−2.28	5.70 × 10^−4^	0.91	15.53	66.9	9.49	[63]
DR3TBDTT-S-E: DR3TBDTT	PC_71_BM	1.73	−5.33/−3.50	6.74 × 10^−4^	0.91	14.89	76.9	10.38	[63]
DCAO3TBDTT: DR3TBDTT-S-E	IDIC	-	-	7.21 × 10^−4^	0.91	16.37	67.4	10.04	[63]
BDT-2T-DCV-Me	IDIC	1.90	−5.56/−3.35	3.48 × 10^−4^	1.06	4.75	29.3	1.56	[64]
BDT-2T-CNAB	IDIC	1.90	−5.54/−3.31	4.67 × 10^−3^	1.04	10.10	58.8	6.17	[64]
BDT-HTOX	PC_71_BM	1.72	−5.44/−3.62	-	0.88	3.26	27.9	0.80	[65]
BDT-TBT	PC_71_BM	2.14	−5.43/−3.29	-	0.84	2.56	31.9	0.69	[66]
BDT-THTBT	PC_71_BM	2.02	−5.33/−3.31	-	0.82	4.20	30.1	1.04	[66]
SD1	Y6T	1.87	−5.08/−3.41	8.32 × 10^−4^	0.88	18.23	63.1	10.12	[67]
SD2	Y6T	1.88	−5.11/−3.41	5.18 × 10^−4^	0.89	17.79	52.3	8.28	[67]
SD3	Y6T	1.89	−5.10/−3.40	3.59 × 10^−4^	0.89	14.47	45.0	5.79	[67]

**Table 3:** Optoelectronic properties and device parameters for BDT-based ASM OSCs via π-bridge engineering.

Donor	Acceptor	E_g_ (eV)	HOMO/LUMO (eV)	*µ*_h_ (cm^2^V^−1^s^−1^)	V_OC_ (V)	J_SC_ (mAcm^−2^)	FF (%)	PCE (%)	Ref.
D1	PC_70_BM	1.61	−5.19/−3.56	2.04 × 10^−4^	1.03	10.07	54.7	5.67	[68]
DO1	PC_70_BM	1.59	−5.18/−3.56	1.71 × 10^−4^	0.91	9.47	48.2	4.15	[68]
D2	PC_70_BM	1.60	−5.16/−3.54	2.82 × 10^−2^	0.92	11.05	66.4	6.75	[68]
DO2	PC_70_BM	1.60	−5.16/−3.52	2.63 × 10^−2^	0.92	8.58	64.8	5.11	[68]
0TBM	PC_71_BM	1.42	−5.13/−4.11	9.06 × 10^−6^	0.64	0.10	31.5	0.02	[69]
1TBM	PC_71_BM	1.46	−5.11/−3.84	6.70 × 10^−5^	0.83	3.49	33.2	0.95	[69]
2TBM	PC_71_BM	1.46	−5.10/−3.80	2.37 × 10^−5^	0.79	6.66	35.1	1.85	[69]
2TBM (out)	PC_71_BM	1.48	−5.09/−3.75	2.37 × 10^−5^	0.81	6.33	30.4	1.56	[69]
3TBM	PC_71_BM	1.42	−5.10/−3.75	7.74 × 10^−3^	0.80	13.81	56.4	6.29	[69]
4TBM	PC_71_BM	1.42	−5.06/−3.76	1.27 × 10^−5^	0.78	5.01	40.3	1.56	[69]
4TBM out)	PC_71_BM	1.56	−5.12/−3.76	1.27 × 10^−5^	0.76	2.33	25.3	0.45	[69]
5TBM	PC_71_BM	1.42	−5.10/−3.74	4.00 × 10^−3^	0.81	9.62	68.8	5.35	[69]
TBDT-2HT-ID	PC_71_BM	1.78	−5.47/−3.68	7.6 × 10^−5^	1.07	12.4	55.5	7.36	[70]
BDT-1T-ID	PC_71_BM	1.80	−5.23/−3.43	1.7 × 10^−3^	1.06	11.0	49.0	5.9	[71]
BDT-2T-ID	PC_71_BM	1.72	−5.13/−3.41	7.1 × 10^−3^	0.96	14.0	49.0	6.9	[71]
BDT-3T-ID	PC_71_BM	1.65	−5.03/−3.38	4.2 × 10^−3^	0.93	13.5	49.0	6.2	[71]
BDT-4T-ID	PC_71_BM	1.64	−4.99/−3.35	5.4 × 10^−3^	0.88	11.3	51.0	5.1	[71]
BDTTID	PC_70_BM	1.74	−5.36/−3.59	1.96 × 10^−5^	1.03	10.20	53.0	5.54	[72]
BDT3TID	PC_70_BM	1.67	−5.16/−3.48	1.87 × 10^−5^	0.89	9.04	59.0	4.74	[72]
BDT(ThBTTh)_2_	PC_61_BM	1.77	−5.17/−3.40	4.7 × 10^−4^	0.89	9.33	54.5	4.53	[73]
BDT(BTTh)_2_	PC_61_BM	1.77	−5.11/−3.34	0.86 × 10^−4^	0.82	4.74	40.5	1.58	[73]
a-SM1	PC_61_BM	1.66	−5.15/−3.49	9.2 × 10^−4^	0.67	4.13	50.1	1.40	[74]
a-SM2	PC_61_BM	1.66	−5.13/−3.47	1.29 × 10^−3^	0.65	6.50	60.3	2.57	[74]
SM-BT-2OR	IDIC	1.77	−5.34/−3.12	7.37 × 10^−5^	0.94	13.57	56.5	7.20	[75]
SM-BT-2F	IDIC	1.66	−5.36/−3.26	1.77 × 10^−5^	0.98	6.74	41.7	2.76	[75]
BDTSe-TTPD	PC_71_BM	1.86	−5.34/−3.48	3.04 × 10^−6^	0.90	10.5	46.3	4.37	[76]
M7a	PC_71_BM	1.84	−5.01/−3.36	-	0.98	7.74	51.0	3.9	[77]
M7b	PC_71_BM	1.88	−5.12/−3.26	-	0.97	5.54	49.0	2.5	[77]
b-SM1	PC_71_BM	1.84	−5.32/−3.16	9.89 × 10^−5^	0.99	11.18	56.0	6.20	[78]
b-SM2	PC_71_BM	1.72	−5.28/−3.24	1.89 × 10^−4^	1.04	12.06	60.0	7.45	[78]
BDT(TVT-SR)_2_	IDIC	1.82	−5.33/−3.18	1.48 × 10^−4^	0.98	15.92	71.2	11.10	[79]
SBDT-BDD	IDIC	1.77	−5.25/−3.55	3.5 × 10^−4^	0.97	15.15	62.5	9.2	[80]
SBDT-BDD	PC_71_BM:IDIC	-	-	3.8 × 10^−4^	0.97	16.21	69.3	10.9	[80]
BDTTNTTR	PC_71_BM	1.51	−5.29/−3.53	2.01 × 10^−3^	0.89	15.70	71.7	10.02	[81]
BDTSTNTTR	PC_71_BM	1.50	−5.35/−3.60	3.18 × 10^−3^	0.93	16.21	76.5	11.53	[81]
H11	IDIC	1.87	−5.31/−3.03	7.7 × 10^−5^	0.98	15.21	65.5	9.73	[82]
H12	IDIC	1.87	−5.28/−3.01	7.9 × 10^−5^	0.96	10.51	54.9	5.51	[82]
H13	IDIC-4F	1.93	−5.43/−3.39	2.02 × 10^−4^	0.94	17.3	63.2	10.3	[83]
H14	IDIC-4F	1.94	−5.46/−3.47	4.41 × 10^−4^	0.94	18.3	70.2	12.1	[83]
BDT(TTzT)_2_	PC_71_BM	1.91	−5.51/−3.67	8.76 × 10^−5^	0.98	9.56	53.5	5.01	[84]
BDT(TTz2T)_2_	PC_71_BM	1.77	−5.30/−3.64	2.14 × 10^−4^	0.88	10.55	57.2	5.29	[84]
TBDT(TTzT)_2_	PC_71_BM	1.99	−5.59/−3.69	4.63 × 10^−4^	1.03	9.50	61.6	6.10	[85]
TBDT(TTz2T)_2_	PC_71_BM	1.82	−5.37/−3.65	7.25 × 10^−4^	0.94	10.65	65.1	6.56	[85]
BDTQ-BDT(EH)	PC_70_BM	2.10	−5.36/−3.26	-	0.83	4.50	32.0	1.20	[86]
BDTQ-BDT(OC)	PC_70_BM	2.11	−5.30/−3.19	-	0.79	3.52	30.0	0.83	[86]
SM-0F	PC_71_BM	1.56^cv^	−5.09/−3.53	2.10 × 10^−4^	0.73	7.3	43.4	2.56	[87]
SM-2F	PC_71_BM	1.49^cv^	−5.12/−3.63	3.52 × 10^−4^	0.75	11.0	44.9	3.94	[87]
SM-4F	PC_71_BM	1.55^cv^	−5.13/−3.58	2.35 × 10^−4^	0.77	9.1	46.7	3.48	[87]
BBDDR	IDIC	1.79	−5.40/−3.61	2.4 × 10^−4^	1.01	14.6	53.0	7.8	[88]
BDT-TITRh	PC_71_BM	1.78	−5.24/−3.74	2.1 × 10^−5^	0.80	13.05	33.8	3.52	[89]
BDT-TI2TRh	PC_71_BM	1.75	−5.17/−3.71	8.0 × 10^−5^	0.85	13.23	37.3	4.19	[89]
BDT-BTF	PC_71_BM	1.78	−5.20/−3.24	3.9 × 10^−3^	0.85	10.48	66.0	5.88	[90]
BDTDPTz	PC_71_BM	1.65	−5.42/−3.65	8.20 × 10^−5^	0.87	12.83	56.4	6.28	[91]
B2TPR	PC_71_BM	1.68	−5.23/−3.55	1.26 × 10^−4^	0.98	11.3	64.0	7.1	[92]
BTRO	IDIC-4F	1.79	−5.30/−3.41	4.29 × 10^−4^	0.91	11.04	41.0	4.08	[93]
BTCN	IDIC-4F	1.82	−5.31/−3.43	6.94 × 10^−4^	0.89	11.46	45.0	4.62	[93]
ECTBD	Y6	1.93	−5.47/−3.54	-	0.81	6.37	30.5	1.58	[94]
ECTBD(15%):PM6	Y6	-	-	-	0.85	25.54	76.2	16.51	[94]
BER6	IDIC	1.91	−5.41/−2.90	1.47 × 10^−4^	0.97	14.86	63.0	9.03	[95]
BECN	IDIC	1.85	−5.44/−2.99	6.44 × 10^−5^	0.96	11.10	51.0	5.52	[95]
D18-Cl:G17(0.9:0.1)	Y6	1.80	−5.39/−3.60	2.53 × 10^−4^	0.88	25.99	76.7	17.13	[96]
D18-Cl:G19(0.9:0.1)	Y6	1.83	−5.30/−3.53	2.77 × 10^−4^	0.87	27.36	77.7	18.53	[96]
SM-BDT	Y8	1.84	−5.10/−2.70	2.04 × 10^−4^	0.84	21.63	58.7	10.68	[97]
SMBDT-S	PC_71_BM	1.85	−5.56/−3.56	-	1.05	5.55	50.0	2.89	[98]
SM-BDT-SF	PC_71_BM	1.86	−5.72/−3.59	-	1.18	1.39	55.0	0.90	[98]

**Table 4:** Optoelectronic properties and device parameters for BDT-based ASM OSCs with various combinations.

Donor	Acceptor	E_g_ (eV)	HOMO/LUMO (eV)	*µ*_h_ (cm^2^V^−1^s^−1^)	V_OC_ (V)	J_SC_ (mAcm^−2^)	FF (%)	PCE (%)	Ref.
BDT-_1_	PC_71_BM	1.77	−5.14/−3.37	5.38 × 10^−5^	0.89	13.02	62.0	7.18	[99]
BDT-_2_	PC_71_BM	1.76	−5.13/−3.37	2.44 × 10^−4^	0.89	13.17	73.0	8.56	[99]
BDT-_3_	PC_71_BM	1.82	−5.10/−3.28	9.19 × 10^−5^	0.90	11.34	70.0	7.14	[99]
BDT_x_-2TVTDPP	PC_61_BM	1.61	−5.10/−3.32	1.61 × 10^−4^	0.67	3.61	65.8	1.58	[100]
BDT_y_-2TVTDPP	PC_71_BM	1.71	−5.30/−3.32	2.8 × 10^−5^	0.86	8.60	38.7	2.85	[100]
aBDT	PC_61_BM	1.96	−5.23/−3.39	3.5 × 10^−5^	0.81	1.74	27.0	0.40	[101]
BDT	PC_61_BM	1.85	−5.16/−3.40	2.7 × 10^−4^	0.85	8.67	49.0	3.60	[101]
B-BDP	PC_71_BM	1.46	−5.11/−3.65	4.53 × 10^−4^	0.73	11.84	53.8	4.65	[102]
PH	PC_61_BM	1.70	−5.36/−3.66	7.45 × 10^−6^	0.83	8.36	59.6	4.15	[103]
PF_2_	PC_61_BM	1.70	−5.47/−3.75	4.01 × 10^−6^	0.94	7.81	57.8	4.26	[103]
PH:PF_2_ (0.1:0.9)	PC_61_BM	-	-	7.17 × 10^−6^	0.93	9.18	57.6	4.90	[103]
DRTB-T-C_2_	IT-4F	2.0	−5.51/−3.34	3.27 × 10^−5^	0.89	16.66	64.0	9.52	[104]
DRTB-T-C_4_	IT-4F	1.99	−5.50/−3.32	1.74 × 10^−5^	0.91	18.27	68.0	11.24	[104]
DRTB-T-C_6_	IT-4F	1.98	−5.50/−3.32	5.55 × 10^−5^	0.93	17.92	63.0	10.52	[104]
DRTB-T-C_8_	IT-4F	1.97	−5.52/−3.33	3.14 × 10^−5^	0.93	16.15	61.0	9.14	[104]
DRTB-O	PC_71_BM	1.90	−5.50/−3.56	5.44 × 10^−5^	1.01	7.49	65.0	4.91	[105]
DRTB-T	PC_71_BM	1.90	−5.48/−3.56	1.14 × 10^−4^	1.01	10.02	70.0	7.08	[105]
DRTB-O	IDIC	-	-	3.74 × 10^−7^	0.99	0.57	27.0	0.15	[105]
DRTB-T	IDIC	-	-	3.46 × 10^−4^	0.98	14.22	65.0	9.06	[105]
DRTB-FT	F-2Cl	1.99	−5.64/−3.61	8.56 × 10^−5^	1.07	13.46	53.2	7.66	[106]
BDT(DPP-9-BTI)_2_	PC_71_BM	1.46	−5.07/−3.61	2.06 × 10^−4^	0.55	11.84	54.1	3.52	[107]
BDT(DPP-8-BTI)_2_	PC_71_BM	1.43	−5.20/−3.77	2.19 × 10^−4^	0.64	11.91	63.4	4.80	[107]
SeBDT-DPP	PC_71_BM	1.63	−5.42/−3.79	5.9 × 10^−3^	0.79	10.98	58.0	5.04	[108]
BDT(DPP)_4_	PC_71_BM	1.69	−5.36/−3.33	-	0.75	8.54	39.0	2.5	[109]
BDT(DPP)_4_	C8-ITIC	-	-	-	0.86	10.1	45.0	3.9	[109]
M1	PC_61_BM	2.31	−5.79/−3.49	-	0.55	3.07	32.0	0.54	[110]
M2	PC_61_BM	2.10	−5.66/−3.52	-	0.61	2.10	32.0	0.41	[110]
c-SM1	ITIC-4F	1.79	−5.37/−3.58	-	0.83	2.28	21.0	0.41	[111]
c-SM2	ITIC-4F	1.64	−5.50/−3.86	-	0.71	4.77	24.0	0.82	[111]
C8T-BDTDP	6TIC	1.58	−5.28/−3.70	1.66 × 10^−4^	0.75	17.75	64.0	8.73	[112]
C8ST-BDTDP	6TIC	1.59	−5.24/−3.65	3.82 × 10^−4^	0.79	19.53	65.6	10.39	[112]
C8TEBDT-2P	IDIC	1.57	−5.24/−3.67	1.26 × 10^−4^	0.86	14.5	64.6	7.46	[113]
C8TBDT-2P	IDIC	1.72	−5.19/−3.47	3.75 × 10^−5^	0.80	6.83	45.8	2.68	[113]
BDT-Qx	PC_71_BM	2.08	−5.34/−3.26	-	0.75	2.20	31.4	0.52	[114]
BDT-T-Qx	PC_71_BM	1.95	−5.35/−3.40	-	0.78	2.41	31.7	0.59	[114]
3BDTBDD	ITIC	2.03	−5.50/−3.38	1.73 × 10^−5^	0.90	9.51	50.6	4.33	[115]
5BDTBDD	ITIC	1.87	−5.46/−3.51	1.12 × 10^−4^	0.91	13.23	65.6	7.89	[115]
3BDT-4	Y6	1.83	−5.14/−3.44	8.0 × 10^−5^	0.83	17.0	41.3	5.82	[116]
3BDT-5	Y6	1.90	−5.15/−3.40	3.64 × 10^−4^	0.84	21.3	58.1	10.4	[116]
O-BDTdFBT	PC_71_BM	1.83	−5.37/−3.52	3.1 × 10^−5^	0.97	11.48	70.0	8.10	[117]
BDT-O-DPP	PC_61_BM	1.69	−5.16/−3.47	1.54 × 10^−4^	0.88	9.54	51.1	4.28	[118]
BDT-PO-DPP	PC_61_BM	1.70	−5.25/−3.55	2.98 × 10^−4^	0.83	11.23	60.3	5.63	[118]
BDT(DPP-TTHex)_2_	PC_71_BM	1.49	−5.16/−3.64	1.01 × 10^−5^	0.65	6.08	60.0	2.36	[119]
BDT(DPP-TT)_2_	PC_71_BM	1.53	−5.15/−3.61	1.28 × 10^−5^	0.69	13.39	56.0	5.12	[119]
BDT(DPP)_2_	PC_61_BM	1.55	−5.62/−3.47	-	0.81	3.20	49.2	1.26	[120]
BDTT(DPP)_2_	PC_61_BM	1.52	−5.68/−3.46	-	0.78	2.83	34.9	0.77	[120]
BDT(TPD-DPP)_2_	PC_61_BM	1.55	−5.67/−3.63	-	0.78	5.69	54.5	2.41	[120]
BDTT(TPD-DPP)_2_	PC_61_BM	1.55	−5.68/−3.64	-	0.77	10.83	50.9	4.25	[120]
TBCA-C2	IT-4F	2.03	−5.51/−3.48	1.52 × 10^−5^	0.91	13.61	59.0	7.34	[121]
TBCA-C4	IT-4F	2.03	−5.49/−3.46	1.06 × 10^−4^	0.93	15.43	64.0	9.21	[121]
TBCA-C6	IT-4F	2.03	−5.53/−3.50	2.42 × 10^−5^	0.94	13.97	60.0	7.91	[121]
TBCA-C8	IT-4F	2.03	−5.52/−3.49	1.38 × 10^−5^	0.94	13.52	57.0	7.24	[121]
BDT-3Th	Y6	1.97	−5.04/−3.39	-	0.87	11.90	36.5	3.78	[122]
BDT-4Th	Y6	2.01	−5.14/−3.38	-	0.84	17.20	40.3	5.83	[122]
BSCl-C1	IDIC-4Cl	1.87	−5.59/−2.25	4.17 × 10^−5^	0.56	4.90	33.9	0.90	[123]
BSCl-C2	IDIC-4Cl	1.82	−5.58/−2.19	3.83 × 10^−4^	0.86	20.10	71.3	12.40	[123]
BSCl-C3	IDIC-4Cl	1.84	−5.55/−2.18	1.58 × 10^−4^	0.87	14.20	67.0	8.25	[123]
BSCl	IDIC-4Cl	1.58	−5.55/−3.30	5.4 × 10^−5^	0.86	21.5	70.0	13.03	[124]

## Data Availability

Not applicable.
